# A first generation inhibitor of human Greatwall kinase, enabled by structural and functional characterisation of a minimal kinase domain construct

**DOI:** 10.18632/oncotarget.11511

**Published:** 2016-08-22

**Authors:** Cory A. Ocasio, Mohan B. Rajasekaran, Sarah Walker, Darren Le Grand, John Spencer, Frances M.G. Pearl, Simon E. Ward, Velibor Savic, Laurence H. Pearl, Helfrid Hochegger, Antony W. Oliver

**Affiliations:** ^1^ Genome Damage and Stability Centre, School of Life Sciences, University of Sussex, Falmer, Brighton, UK; ^2^ Cancer Research UK DNA Repair Enzymes Group, Genome Damage and Stability Centre, School of Life Sciences, University of Sussex, Falmer, Brighton, UK; ^3^ Sussex Drug Discovery Centre, School of Life Sciences, University of Sussex, Falmer, Brighton, UK; ^4^ School of Life Sciences, University of Sussex, Falmer, Brighton, UK; ^5^ Brighton and Sussex Medical School, University of Sussex, Falmer, Brighton, UK

**Keywords:** kinase, inhibitor, Greatwall, ENSA, cancer

## Abstract

MASTL (microtubule-associated serine/threonine kinase-like), more commonly known as Greatwall (GWL), has been proposed as a novel cancer therapy target. GWL plays a crucial role in mitotic progression, via its known substrates ENSA/ARPP19, which when phosphorylated inactivate PP2A/B55 phosphatase. When over-expressed in breast cancer, GWL induces oncogenic properties such as transformation and invasiveness. Conversely, down-regulation of GWL selectively sensitises tumour cells to chemotherapy. Here we describe the first structure of the GWL minimal kinase domain and development of a small-molecule inhibitor GKI-1 (Greatwall Kinase Inhibitor-1). *In vitro*, GKI-1 inhibits full-length human GWL, and shows cellular efficacy. Treatment of HeLa cells with GKI-1 reduces ENSA/ARPP19 phosphorylation levels, such that they are comparable to those obtained by siRNA depletion of GWL; resulting in a decrease in mitotic events, mitotic arrest/cell death and cytokinesis failure. Furthermore, GKI-1 will be a useful starting point for the development of more potent and selective GWL inhibitors.

## INTRODUCTION

The mitotic kinase MASTL (microtubule-associated serine/threonine kinase-like) - more commonly known as Greatwall kinase or GWL - belongs to the AGC family of serine/threonine protein kinases and has recently emerged as a potential target for cancer chemoprevention [1–5]. GWL exerts its biological activity by phosphorylating α-endosulfine (ENSA) and/or cAMP-regulated phosphoprotein 19 (ARPP19). Once phosphorylated, these proteins become potent inhibitors of the protein phosphatase 2A complex (PP2A/B55) [6, 7]. PP2A/B55 counteracts CDK1 during mitosis by dephosphorylating mitotic CDK1 substrates. Negative regulation of PP2A/B55 by GWL thus results in a positive feedback loop that boosts CDK1 activity above the threshold required for mitotic entry. In addition to its mitotic roles, GWL also contributes to AKT activation by negatively regulating the AKT phosphatase PHLPP [5]. Moreover, studies from both budding and fission yeast have also implicated the GWL/ENSA pathway in linking metabolic responses to cell cycle control [8–10].

Mutation of GWL in *Drosophila* causes defects in chromosomal condensation, as well as delayed mitotic entry and exit in neuroblasts [11]. Studies in *Xenopus* extracts also showed that GWL plays an important role in mitotic entry and in DNA-damage checkpoint recovery in late G2-phase [12–14]. Likewise, depletion of the mammalian orthologue of GWL by siRNA in human cells, or by Cre-mediated excision in mouse embryonic fibroblasts, causes severe mitotic phenotypes such as aneuploidy, defects in chromosome condensation and failure to inactivate the spindle assembly checkpoint, with consequent defects in chromosome segregation and cytokinesis [1, 15–17].

Non-transformed cell lines, such as HaCaT and OKF4, have been found to have significantly lower levels of GWL protein when compared to some cancer-derived cells lines [4]. Accordingly, levels of GWL are seen to be elevated in oral squamous cell carcinoma, breast cancer and prostate cancer tissues [5]. Taken together these findings suggest that GWL may have an adaptive role in some cancer types, and may contribute directly to tumourigenesis. This could potentially be due to the non-mitotic functions of GWL, such as the regulation of AKT activity, but the precise role of GWL in cellular transformation remains largely unexplored. Thus, pharmacologic targeting of GWL could be a useful tool for analysing these tumour-associated functions and may ultimately prove to be a clinically useful strategy for targeting specific sub-classes of tumours.

To date, no X-ray structures of GWL are available. Although highly related at the amino acid sequence level to the N- and C-terminal kinase lobes of the MAST kinases (microtubule-associated serine/threonine kinase; MAST1, 2, 3 and 4) and other AGC kinases, GWL has a highly unusual architecture, with an ~500 amino acid insertion between the DFG and APE motifs of the activation segment connecting the N- and C-terminal lobes (NCMR; non-conserved middle region). The structure and function of the NCMR, which is less well conserved than the encompassing kinase domain, remains enigmatic, but appears to be dispensable, at least in part, for its biological function [18].

In pursuit of developing human GWL as a target for drug discovery we have developed a minimal GWL kinase domain construct, in which the NCMR has been deleted and a conventional AGC-kinase activation segment inserted in its place. This construct is soluble even when purified from *E.coli*, and displays specific kinase activity towards its *bona fide* substrate ENSA. We have determined the X-ray crystal structure of this construct and have developed a first generation inhibitor displaying *in cellulo* efficacy, based on a small-scale inhibitor screen and rational SAR-driven design. This molecule, GKI-1, may find utility as a lead / tool compound to inform the on-going development of potent and specific GWL inhibitors.

## RESULTS

### Construct design, expression and purification

**Figure 1:**
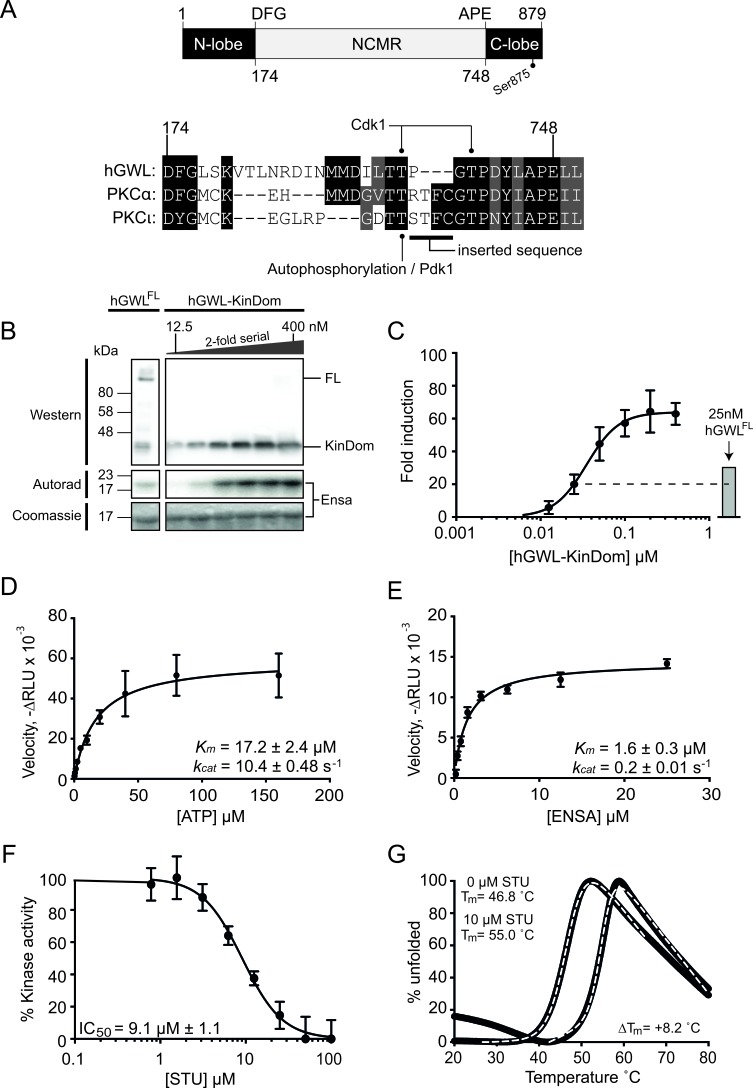
Biochemical and biophysical characterisation of the minimal kinase construct hGWL-KinDom (**A.**, top) GWL is an unusual bifurcated protein kinase, containing a large ~500 amino acid insertion (NCMR, non-conserved middle region) between the conserved DFG and APE motifs of the activation segment / T-loop. The amino acid boundaries of the N- and C-lobes of the kinase domain are indicated, as well as the C-terminal phosphorylation site (Ser875) known to be essential for function. (**A.**, bottom) Amino acid sequence alignment of the activation segment / T-loop of human GWL with the PKC isoforms alpha (α) and iota (ι). Threonine residues within this region of GWL are known to be phosphorylated *in vivo*, Thr194 by Cdk1 [28] and the equivalent of Thr718 in *Xenopus laevis* GWL (T725) [24]. The equivalent residues in PKC-α and PCKι, Thr497 and Thr412 respectively, are instead phosphorylated by PDK1. The ‘RTFC’ sequence taken from PKCα, in order to generate hGWL-KinDom is highlighted. Absolutely conserved residues are shown with a black background, with residues conserved by property shown with a grey background. **B.** hGWL-KinDom was serially diluted by 2-fold and each concentration was subjected to a radioactive kinase assay. The same concentrations of hGWL-KinDom were analysed by western analysis. Immunoprecipitated Flag-tagged hGWL^FL^ was subjected to the same analyses as assay controls. **C.** Kinase activity was normalised to total ENSA levels, measured as the ^32^P-Ensa intensity (autorad)/ENSA intensity (densitometry) ratio and plotted as fold-induction relative to background; kinase deficient control reaction. (D, E) Kinetic parameters (k_cat_, K_M_) were established by diluting either ATP or ENSA serially by 2-fold and subjecting each concentration to a Kinase-Glo Max assay. Non-linear regression using Prism 6.0 and the built-in enzyme kinetics module were used to determine Michaelis-Menten kinetic parameters, *k*_cat_ and *K*_m_, for both ATP **D.** and ENSA **E. F.** hGWL-KinDom was subjected to either increasing concentrations of staurosporine (STU) or DMSO and the resulting kinase activity was determined using the Kinase-Glo Max assay. The % kinase activity was normalised to the DMSO control and non-linear regression using Prism 6.0 was used to determine IC_50_ values. **G.** Thermal shift assay. Binding of hGWL-KinDom (1.5 μM) to STU (10μM) produces a positive temperature mid-point (T_m_) shift of 8.2°C, indicating compound binding.

We found that recombinant full-length human GWL (hGWL^FL^) containing the entire NCMR insert (Figure 1A, Top) did not express in a soluble form in *Escherichia coli*. Although amenable to expression in *Spodoptera frugipera* cells using recombinant baculovirus, the overall yields were low (our own unpublished observations). We therefore sought an alternative strategy to produce enough protein to facilitate both structural and functional studies, as well as to support our drug discovery efforts.

Through bioinformatic analysis, we noted that the amino acid sequence preceding the conserved ‘APE’ kinase motif of hGWL resembled that of the PKC (Protein Kinase C) family of kinases, in particular the alpha isozyme [19] (Figure 1A, Bottom). We therefore excised the NCMR of hGWL and replaced it with four amino acids 496 - RTFC - 499 taken from human PKCα, to generate the expression construct ‘hGWL-KinDom’ (see Experimental Section).

The resultant fusion was readily expressed in, and then purified from *E.coli,* using standard chromatographic procedures (see Supporting Information). The GST-hGWL-KinDom fusion co-purified with the *E.coli* chaperone GroEL, but could be efficiently released by incubation with ATP [20] and the affinity tag removed, to give pure soluble hGWL-KinDom (Supporting Information, Figure S1).

### Enzyme characterisation

Firstly, we examined the phosphorylation status of purified hGWL-KinDom by mass spectrometry. We could identify peptides, obtained from protease digests, which corresponded to phosphorylation at amino acids Thr17, Thr868, Thr873, Ser875 and Ser878 (Supporting Information, Figure S2). Experiments with immunoprecipitated full-length GWL (human or *Xenopus*), as well as several independent proteomic screens, have documented both Ser875 and Ser878 phosphorylation sites [18, 21–24]. The three remaining sites are therefore likely to be unique to hGWL-KinDom and arise as a direct consequence of its heterologous expression in *E.coli*; an observation consistent with other human protein kinases [25]. Arguably however, only phosphorylation at Ser875 is functionally relevant - the S875A mutation severely compromises GWL kinase activity, whereas the S878A mutation does not [18, 24]. Mutation of Ser875 also prevents the timely reactivation of PP2A activity in order to complete mitosis [26].

We next sought to test if hGWL-KinDom phosphorylated the known GWL substrate ENSA [6, 7, 27]. To this end, we used an *in vitro* kinase reaction with radiolabelled ATP (g-^32^P) to directly visualise phosphorylated ENSA by autoradiography (Figure 1B) and then to quantitate activity using photo-stimulated luminescence (Figure 1C). The enzyme readily phosphorylated ENSA, and at high concentrations reached saturation. Flag-tagged hGWL^FL^ immunoprecipitated from human cells was used as an assay control [28] and displayed only slightly greater activity when compared to the equivalent concentration of hGWL-KinDom (25 nM; Figure 1B and 1C).

Secondly, using a commercial luminescence-based assay (Kinase-Glo Max, Promega), we were subsequently able to measure enzymatic parameters for hGWL-KinDom determining *K_m_* and *k_cat_* values of 17.2 ± 2.4 μM and 10.4 ± 0.48 s^−1^ for ATP hydrolysis, and *K_m_* and *k_cat_* values of 1.6 ± 0.3 μM and 0.2 ± 0.01 s^−1^ for ENSA as a substrate (Figure 1D and 1E).

We next tested if the pan-kinase inhibitor staurosporine (STU) affected hGWL-KinDom catalytic activity by incubating fixed concentrations of the enzyme, ENSA and ATP with increasing concentrations of the inhibitor, and then assaying for ATP turnover in the same luminescence-based format. STU clearly inhibited kinase activity, with an IC_50_ of ~ 9 μM (Figure 1F). We additionally confirmed binding of STU to hGWL-KinDom by thermal shift assay [29] (Figure 1G), producing a robust temperature midpoint shift (ΔT_m_) of 8.2°C under the experimental conditions tested (Supporting Information).

### Crystal structure of hGWL-KinDom in complex with staurosporine

**Figure 2:**
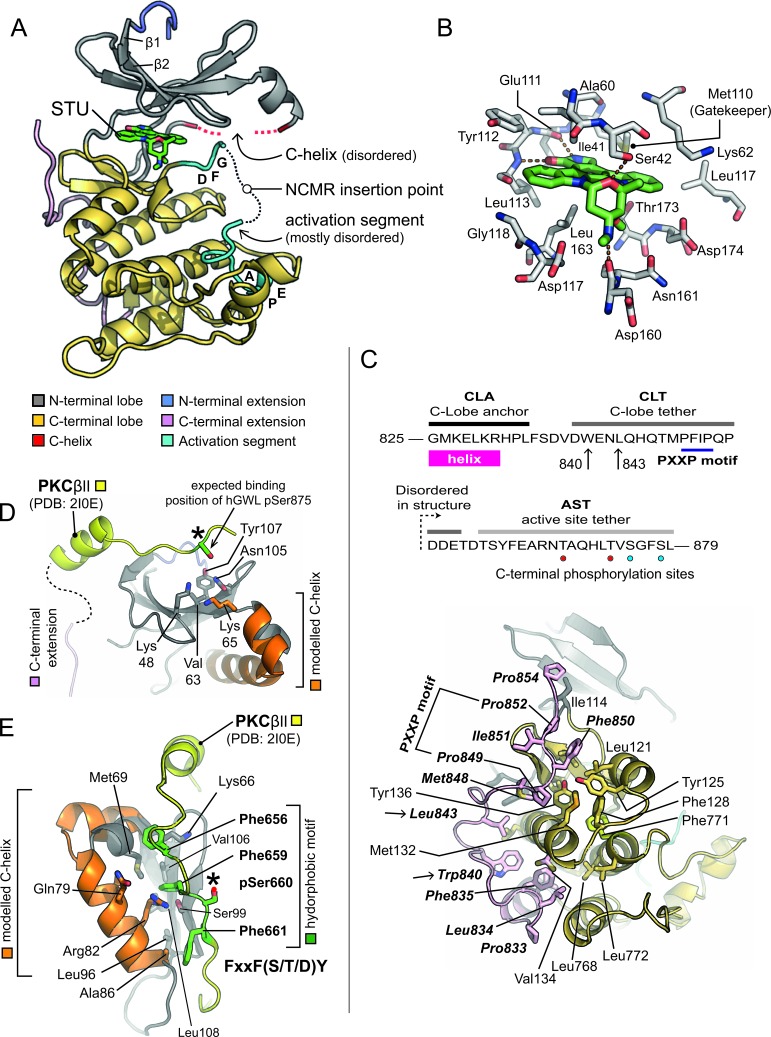
X-ray crystal structure of hGWL-KinDom in complex with STU **A.** Secondary structure molecular cartoon. The components of the kinase domain are coloured according to [30]. Staurosporine (STU) is shown in stick representation, with carbon atoms coloured in green. Please see associated key for further details. **B.** Molecular details for STU bound to hGWL-KinDom. Selected amino acids are shown in stick representation (labelled), with carbon atoms coloured in grey. The carbon atoms of STU are coloured in green. Orange dotted-lines indicate potential hydrogen-bonds made between the protein and STU. (**C.**, Top) Amino acid sequence for the C-terminal region of GWL (amino acids 825-869), highlighting the C-lobe anchor (CLA), C-lobe tether (CLT) and active-site tether (AST) regions; as described in [24]. The positions of Trp840 and Leu843, residues which when mutated affect GWL kinase activity, are indicated (arrows). Phosphorylation sites, unique to the AST of hGWL-KinDom when expressed in *E.coli*, are indicated by red coloured circles. Sites common to both hGWL^FL^ and hGWL-KinDom are indicated by cyan coloured circles. (**C.**, Bottom) Molecular details for the CLA/CLT region of hGWL-KinDom. Amino acids of the CLA/CLT regions (coloured in pink) make a series of predominantly hydrophobic interactions with the C-lobe of the kinase domain (coloured yellow). The identity of key amino acid residues are indicated (stick representation). The positions of Trp840 and Leu843 are additionally highlighted (arrows). **D.** Molecular details for the phospho-binding pocket of GWL. Using the X-ray crystal structure of the PKCβ II kinase domain as a reference (PDB: 2I0E), it is possible to determine that amino acids Lys48, Lys65 and Tyr107 (stick representation) are positioned such that they could interact with the phosphorylated AST of GWL (pSer875). **E.** Molecular details for the Hydrophobic Motif-binding pocket. The side chains of amino acids Lys66, Met69, Gln79, Arg82, Ala86, Leu90, Leu108, and Phe659 (stick representation) form a potential hydrophobic motif-binding pocket in the N-lobe of GWL. Please see associated key for additional details. Note, in Figures D and E, the C-lobe of hGWL has been homology-modelled (carbons coloured in orange).

Diffracting crystals of hGWL-KinDom in complex with STU were obtained from commercially available screens. The structure was determined by molecular replacement, and refined at a resolution of 3.1Å (see Supporting Information, Figure S3 and Table S1 for crystallographic and refinement data). As expected, hGWL-KinDom adopts the classical ‘two-lobe’ protein kinase domain fold [30] (Figure 2A). As both the C-helix and activation loop regions are disordered in electron density maps, this strongly suggests that just the non-phosphorylated form of hGWL-KinDom (in complex with STU) was selectively incorporated into protein crystals.

As expected, STU occupies the nucleotide-binding pocket, making hydrogen bonds to the backbone carbonyl and nitrogen of hinge residues Glu111 and Leu113, via its lactam nitrogen (N6) and oxygen (O6) groups, respectively (nomenclature as described in [31]). It is also ‘sandwiched’ on both sides, by several hydrophobic residues, including Ile41, Val49, Ala60 and Leu163 from the N-lobe, and Thr173 and Leu163 from the C-lobe (Figure 2B).

### The C-terminal extension of hGWL-KinDom

A homology model for the kinase domain of hGWL was previously reported by Vigneron *et al.* [24], which they used to determine if conserved motifs of the ‘C-terminal extension’ - found in many AGC-family protein kinases - were also present in GWL (Figure 2C, top; reviewed by [30, 32, 33]); their analyses confirmed the presence of AGC-insert, C-lobe anchor (CLA), C-lobe tether (CLT) and active-site tether (AST) motifs, and absence of the N-lobe tether (NLT) and hydrophobic (HM) motifs.

In electron density maps, we can observe amino acids 825-854 of the GWL C-terminal extension, containing both the C-lobe anchor (CLA) and the majority of the C-lobe tether (CLT) motifs (Figure 2C, bottom). However, the active-site tether (AST) region at the extreme C-terminus of hGWL-KinDom is not visible (aa 855-879) despite being encoded in the expression construct.

As in other AGC-kinases [30, 32, 33], such as PDK1 [34] and PKCα [35], the GWL C-terminal extension wraps across one face of the C-lobe, making an extensive series of predominantly hydrophobic interactions. A highly conserved aromatic side chain Trp840 and a subsequent hydrophobic residue Leu843, both situated within the CLA and immediately preceding the PXXP motif (849-PFIP-852), serve to anchor the extended C-terminal region to the body of the kinase domain (Figure 2C, top and bottom). Mutation of residues within either motif, when introduced into full-length hGWL results in markedly reduced levels of kinase activity (W840A = 57% reduction, P849A / P852A = 90% reduction; [24]) confirming their relevance and importance to the normal cellular functions of GWL.

Vigneron *et al.,* also indicated that GWL contains both a functional tail / linker binding pocket, and a hydrophobic-motif binding pocket within its N-lobe, which we confirm and corroborate with our structural data. The side-chains of Lys48 (N-lobe) and Lys65 (C-helix) would be suitably positioned to interact with the Ser875 phosphorylation site (AST motif) that is critical for GWL function, as indicated by structural superimposition of the C-terminal extension of PKC βII onto hGWL-KinDom (PDB: 2I0E; Figure 2D). Likewise, a canonical HM from another protein kinase [F-x-x-F-(S/T/D)-Y/F] could be readily accommodated by a pocket lined by the side-chains of Met69, Ser99, Val106 and Leu108 of the N-lobe, plus Gln79 and Arg82 of the C-helix (Figure 2E).

### Pharmacological classification of hGWL-KinDom

With both biochemical and structural validation of our minimal kinase domain, we next sought to pharmacologically characterise hGWL-KinDom, to address its suitability for screens that looked to identify inhibitors of the full-length human enzyme.

Using the same luminescence-based kinase assay, we determined IC_50_ values for inhibition of both hGWL-KinDom and immunoprecipitated hGWL^FL^ kinase activity by STU; 9.1 and 3.1 μM respectively (Figures 1F and Supporting Information, Figure S4A and C). This reasonable level of agreement between values (an ~3-fold difference) indicates that STU has similar affinities for both the minimal kinase domain and its full-length counterpart.

**Figure 3:**
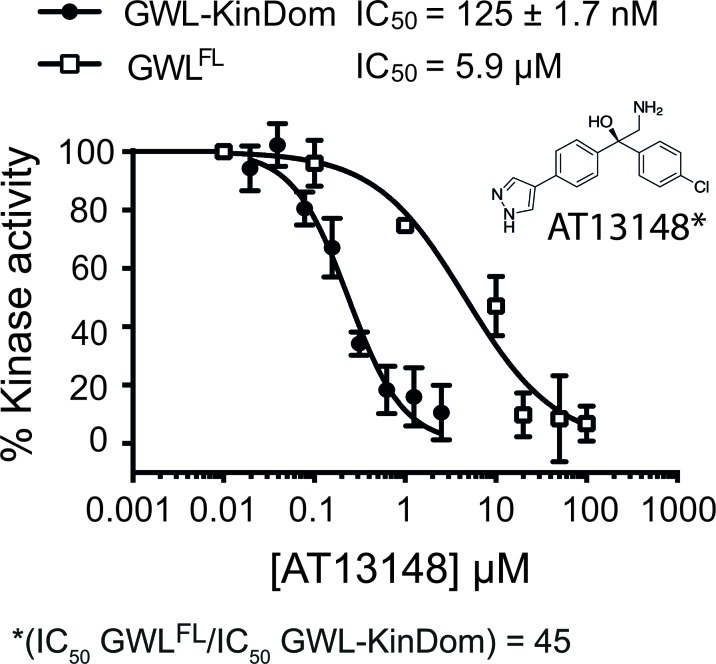
Pharmacologic analysis of hGWL-KinDom and hGWL^FL^ with the AGC kinase inhibitor AT13148 hGWL-KinDom (•) or immunoprecipitated hGWL^FL^ (☐) was treated with increasing concentrations of AT13148 and kinase activity was measured by either the Kinase-Glo Max (•) or immunoprecipitation kinase assays (☐). Non-linear regression with Prism 6.0 was used to determine IC_50_ values.

We next demonstrated that the known AGC kinase inhibitor AT13148 inhibited hGWL-KinDom, determining an IC_50_ of 125 ± 1.7 nM; a value similar to those determined for other AGC kinases inhibited by this compound [36] (Figure 3). Interestingly, when tested on hGWL^FL^ we determined that the IC_50_ values of AT13148 and STU are nearly equipotent at hGWL^FL^ inhibition (AT13148 IC_50_/STU IC_50_ = ~ 2) (Figure 3 and Supporting Information, Figure S4B and D). Together these data suggest that, despite the complete excision of the NCMR insertion, hGWL-KinDom retains several important structural features specific to hGWL>^FL^ (and other AGC kinases) in and around its ATP-binding pocket. With this validation of our experimental approach, we decided to use hGWL-KinDom in screens as part of our discovery efforts to identify inhibitors of GWL kinase activity.

### Structure-activity relationship analysis of ‘hits’

Select compounds, ranking amongst the 8 most potent, identified in a high-throughput screen of a small-molecule library enriched for protein kinase inhibitors (Supporting Information, Figure S5) were taken forward into a second, confirmatory screen, using immunoprecipitated hGWL^FL^ as the enzyme (Supporting Information, Figure S6). Interestingly, GRI-3 and GRI-4 produced IC_50_ values with a good level of agreement to those previously obtained for hGWL-KinDom (Table 1, Supporting Information, Figures S6 and S7). However, both GRI-1 and -2, did not demonstrably inhibit the full-length enzyme (Table 1, Supporting Information, Figure S6A and B). We also found a similar disagreement in IC_50_ using the AGC kinase inhibitor AT13148; inhibitory ratio = 45, (IC_50_ hGWL^FL^ /IC_50_ hGWL-KinDom) (Figure 3).

**Table 1:** Secondary screening of select compounds from the inhibitor screen was performed using Flag-tagged hGWLFL and hGWL-KinDom in the immunoprecipitation and Kinase-Glo Max kinase assays

Structure	Compound	Rank	% Inhibition^1^	IC_50_ (μM)^2^	IC_50_ (μM)^3^
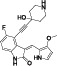	GRI-1	1	67.6	>100	N.D.
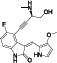	GRI-2	5	37.0	>100	N.D.
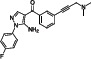	GRI-3*	3	39.6	66	109 ± 13.7
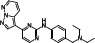	GRI-4**	8	30.1	2.5	3.22 ± 0.31

IC_50_ values were measured for GRI-1, -2, -3 and -4.

1 Screening Data, ATP concentration = 45 μM

2 GWL^FL^

3 GWL-KinDom

* (IC_50_ GWL^FL^/IC_50_ GWL-KinDom) = 0.6,

** (IC_50_ GWL^FL^/IC_50_ GWL-KinDom) = 0.8

**Figure 4:**
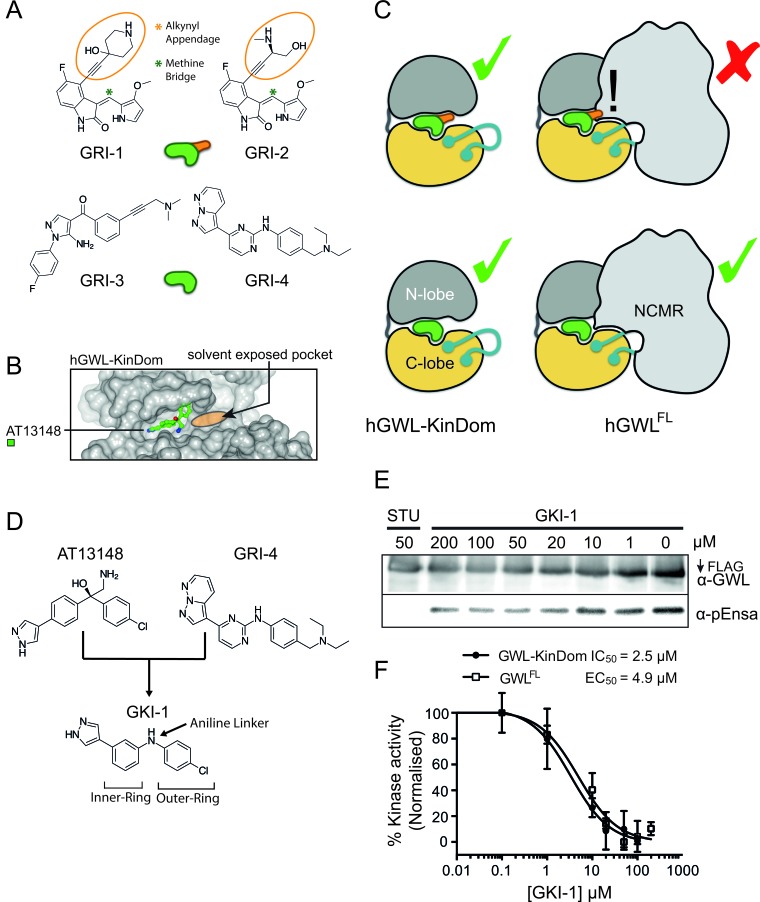
Model describing the minimal structural requirements for small-molecule inhibition of hGWL^FL^ **A.** Small molecule inhibitors are rendered as a green or green and orange cartoons with (orange appendage) or without the alkynyl appendage. **B.** GWL is rendered as a cartoon with (hGWL^FL^) or without (hGWL-KinDom) the NCMR region. This model illustrates how the NCMR could act as a steric filter, which prevents small-molecules with large appendages from docking within the nucleotide-binding site. **C.** Molecular modelling of AT13148 within the nucleotide-binding site of hGWL. Docking with hGWL-KinDom crystal structure (where the disordered C-helix was homology-modelled) reveals a large solvent-exposed pocket vicinal to the ligand-binding site. **D.** Rational design of a first generation hGWL inhibitor using an AT13148 scaffold and incorporating the GRI-4 aniline linker. **E., F.** hGWL-KinDom (•) or immunoprecipitated hGWL^FL^ (•) were treated with increasing concentrations of GKI-1 and kinase activity was measured by either the Kinase-Glo Max (☐) or immunoprecipitation kinase assays (☐). (F) % kinase activity was normalised to the DMSO control, and the smallest and largest values defined and plotted as 0 and 100 % kinase activity respectively. Non-linear regression with Prism 6.0 was used to determine IC_50_ (hGWL-KinDom) or EC_50_ (hGWL^FL^) values.

A common feature of AT13148, GRI-1 and -2 is that they contain branching groups that are connected to either a bridging quaternary carbon (AT13148) or that protrude toward, and are situated directly above the bridging methine linking group (GRI-1 and -2, Figures 3 and 4A). Compounds GRI-3 and -4, whilst showing structural similarity to the AT13148 inhibitor, lack this substitution pattern around their aniline and ketone linkers (Table 1 and Figure 4A).

Superposition of PKA-PKB chimera in complex with AT13148 (PDB: 4AXA) onto hGWL-KinDom (where the disordered C-helix was modelled by sequence-threading [37]) revealed a large, solvent-exposed pocket proximal to the ATP / inhibitor-binding site (Figure 4B). In the minimal kinase domain, this pocket may facilitate binding of all the compounds, whereas in the full-length protein, where the NCMR is likely to occupy (some or all of) this space, GRI-1, -2 and AT13148, would be prevented from binding through steric hindrance (Figure 4B and 4C).

**Scheme 1:**
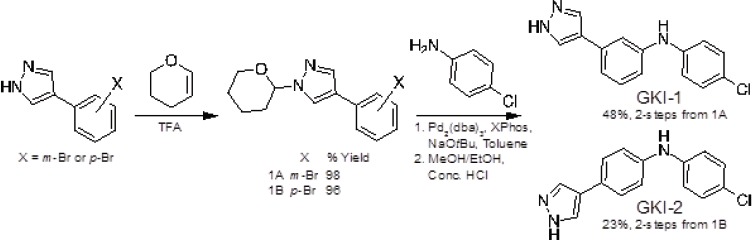
Synthesis of GKI-1/2

To test this hypothesis, we synthesised the ‘hybrid-compounds’ GKI-1 and -2, where we eliminated both the alcohol and methylamine groups of AT13148, and replaced the carbon linker with an aniline linker, to theoretically remove the chemical elements that correlated negatively with inhibition of full-length GWL (Figure 4D, Supporting Information, Figure S8A, Scheme 1). Interestingly, whilst molecular docking studies indicated that both compounds should have similar binding energies (−7.4 to −7.9 kcal/mol; Supporting Information, Figure S8A and B), GKI-2 did not demonstrably inhibit hGWL^FL^ (Supporting Information, Figure S8C and D). It was therefore not taken forward into cellular assays. In contrast, the EC_50_ and IC_50_ values obtained for GKI-1 against both hGWL^FL^ and hGWL-KinDom were found to be highly similar (4.9 and 2.5 μM respectively, inhibitory ratio = ~ 2; Figure 4E and 4F).

We also tested GKI-1 against CDK2 (IC_50_ value of 89.0 ± 2.7 nM, Table 2), as we believed it would have increased specificity towards GWL, over the parent compound GRI-4; a potent CDK inhibitor [38]. As predicted, GKI-1 had no observable inhibitory activity towards CDK2 at concentrations up to 100 μM (Table 2). The X-ray crystal structure of CDK2 in complex with a GRI-4 related compound, indicates that an anilinopyrimidine constituent is important for compound binding, as it participates in hydrogen-bonding interactions with the hinge region (PDB: 3EID [38]). We believe, therefore, that the lack of CDK2 inhibitory activity is due to the absence of this group in GKI-1.

**Table 2:** Specificity profile of GKI-1

	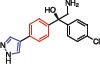 AT13148	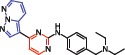 GRI-4	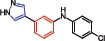 GKI-1
Kinase	IC_50_ (μM ­± S.D.)		
PKA	0.00613 ± 0.002	>100	>40
ROCK1	0.0114 ± 0.004	21.6 ± 5.3	11.3 ± 3.4
CDK2	N.D.	0.0890 ± 0.003	>100

AT13148, GRI-4 and GKI-1 were tested against PKA, ROCK1 and CDK2 using the Kinase-Glo Max and immunoprecipitation kinase assays.

### Selectivity profiling of GKI-1

To determine whether GKI-1 had any level of selectivity towards kinases within the AGC-family, we examined its effect on the activities of two commercially available proteins, ROCK1 and PKA; selected as they are known to be potently inhibited by the AGC kinase inhibitor AT13148, with experimentally determined IC_50_ values of ~6 and 11 nM respectively [36] (Table 2).

GKI-1 robustly inhibited ROCK1 with an IC_50_ of ~11 μM, but only weakly affected PKA; IC_50_ > 40 μM (Table 2). The bioinformatics tool Kinase SARfari (EMBL-EBI) enabled us to rationalise these data, as it indicates that the active site of ROCK1 (at the amino acid sequence level) is more similar to that of hGWL (distance score = 0.85; Supporting Information, Table S2) than those of the PKA isoforms (score = 1.85), thus providing a simple explanation for the apparent selectivity profile of GKI-1 [39]; 2-fold selectivity for binding to hGWL^FL^ over ROCK1, but greater than 8-fold selectivity over PKA.

In order to gain a greater understanding of potential off-target interactions of GKI-1, we undertook more rigorous kinase inhibition profiling (International Centre for Kinase Profiling, University of Dundee) where GKI-1 was tested against 50 different protein kinases, carefully selected to provide representative sampling across the human kinome. At the 25 μM concentration tested, GKI-1 clearly has several off-target interactions, but they are particularly focussed around the AGC kinase family (Supporting Information Figure S9).

### *In cellulo* efficacy of GKI-1

**Figure 5:**
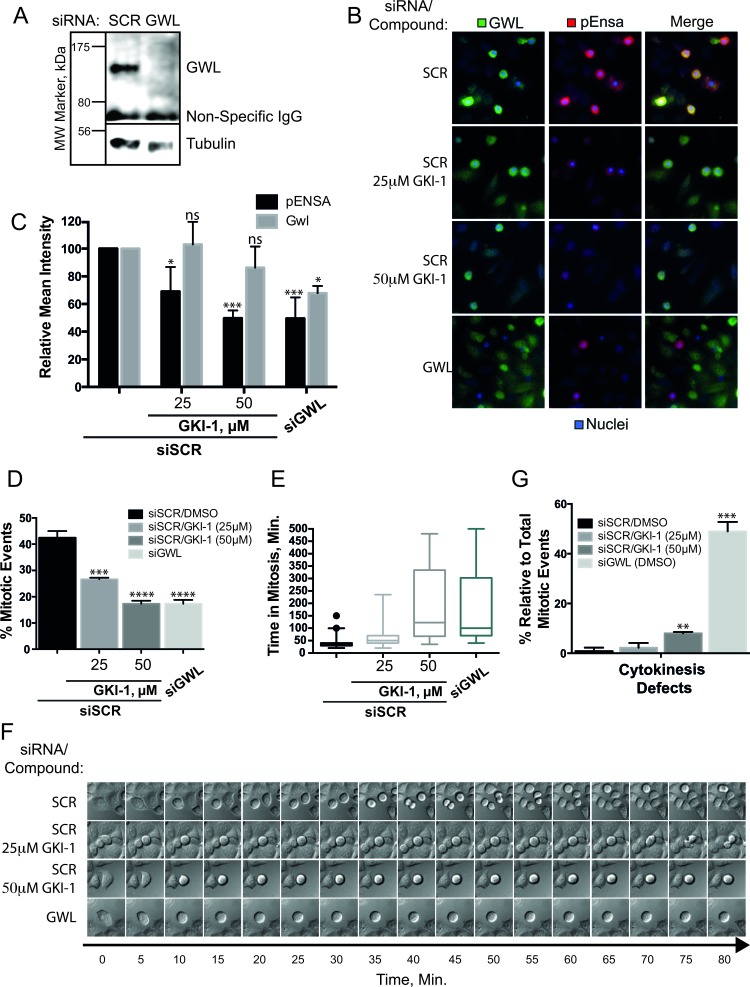
*In cellulo* efficacy of GKI-1 in HeLa cells **A.** HeLa cells were transfected with either non-targeting (siSCR) or GWL targeting siRNAs (siGWL). GWL and loading control protein (Tubulin) were detected by western analysis. **B., C.** HeLa cells treated with siSCR or siGWL (48-h) were arrested in mitosis using nocodazole and treated with DMSO, 25 μM or 50 μM GKI-1. Immunofluorescent staining of p-ENSA (Red) and GWL (Green) was achieved using anti-phospho(Ser67)-ENSA and anti-MASTL(GWL, RIPLY 74C) antibodies and DAPI was used to stain the nucleus (Blue). (C) Quantitation of the IF p-ENSA and GWL signals was performed using the ScanR High-Content Screening Station. The Circularity and Total DAPI parameters were used to identify mitotic cells. **D., E.** HeLa cells treated with siSCR or siGWL (48-h) were treated with DMSO, 25 or 50 μM GKI-1. 4-h after treatment, μ-slides were mounted onto an Olympus IX73 microscope, within a temperature-controlled 37°C chamber maintained at 5% CO_2_, and images were acquired every 5-min for 8.5-h. Time-lapse videos were generated using ImageJ and cellular phenotypes recorded: Mitotic events (D), mitotic arrest (E, F) and failed cytokinesis **G. F.** Kymographs showing an example of typical phenotypes were generated by capturing images every 5 minutes for 80 minutes. Image sequences were chosen to start just before mitotic entry. (B - G) A total of 3 - 5 biological replicates were completed per condition and the t-test statistical module of Prism 6.0 was used to determine p-values (ns (not statistically significant): *P* > 0.05; *: *P* ≤ 0.05; **: *P* ≤ 0.01; ***: *P* ≤ 0.001).

To more directly address the presence of other off-target effects, and / or any level of compound toxicity, we conducted a more comprehensive phenotypic analysis of GKI-1 and GWL siRNA (siGWL, Figure 5A) treated cells by immunofluorescence (IF) and time-lapse video microscopy. Using an antibody that specifically detects phosphorylation of ENSA-Ser67 (p-ENSA) [28], we established an IF assay in order to measure GWL kinase activity in HeLa cells. We treated the cells with nocodazole in order to arrest cells in mitosis, the cell cycle stage where GWL is maximally active [16] (Figure 5B).

Co-incubation of HeLa cells with nocodazole and GKI-1 resulted in a dose-dependent decrease in the levels of p-ENSA as judged by immunofluorescence; an effect mirrored by siRNA depletion of GWL (Figure 5B). Quantitation indicated a 2-fold reduction in p-ENSA levels, compared to control, when cells were treated with 25 μM GKI-1. A concentration of 50 μM GKI-1 further reduced the p-ENSA signal to levels comparable to siGWL treated cells (Figure 5C).

The hallmark phenotypes of GWL depletion in mammalian cells are a delay in mitotic entry, prolonged prometaphase arrest and mitotic exit with aberrant cytokinesis [15, 16]. To test if our compound elicits similar phenotypes we performed live cell imaging of HeLa cells treated with 25 and 50 μM GKI-1. We found that cells treated with GKI-1 were highly sensitive to fluorescent light in the blue to green spectrum, which limited our analysis to differential interference contrast imaging. This is likely due to the photo-stimulated production of cytotoxic GKI-1 metabolites [40]. When compared to cells treated with a scrambled control siRNA (SCR), both siGWL and GKI-1 treatments resulted in a general decrease in mitotic events (Figure 5D and Supporting Information, Videos SV1–SV4) with 50 μM GKI-1 eliciting an effect comparable to siGWL treatment. It is noteworthy that this dose-dependent decrease in mitotic events mirrors the decrease in p-ENSA levels with increasing GKI-1 concentrations.

Furthermore, relative to SCR-treated cells, both siGWL and GKI-1 treatment produced a significant delay in mitotic progression (Figure 5E and 5F, and Supporting Information, Videos SV1–SV4). Cells treated with 50 μM GKI-1 remained in prometaphase/metaphase for two to six hours and exited mitosis with pronounced blebbing and cell death. A proportion of cells underwent successful cell division that often resulted in unevenly sized daughter cells. GWL depletion resulted in a similar mitotic delay but caused a more pronounced failure of cytokinesis as has been previously observed [17] (Figure 5G and Supporting Information, Video SV4).

## DISCUSSION

Our study describes a biochemical workflow that was successfully utilised to enable the facile discovery of a small-molecule inhibitor of human GWL. Through creation of a functional, minimal kinase domain expression construct (hGWL-KinDom) that is readily expressed in *E.coli*, we produced sufficient amounts of recombinant protein in order to support both inhibitor screening and structure-based drug discovery efforts. Whilst some false-positives arose from screens with hGWL-KinDom - due to its expanded ATP-binding pocket, generated as a direct consequence of NCMR deletion - they were identified, and thus eliminated in a secondary, confirmatory screen using immunoprecipitated full-length hGWL.

It is noteworthy that replacing the ~500 residues of the NCMR, with a 4 amino acid AGC-kinase activation segment motif, resulted in a fully active kinase that was capable of phosphorylating its *bona fide* substrate *in vitro*. This indicates that the NCMR does not have an essential role in GWL kinase activity, and leaves the question open as to what the biological role of this unique structural feature actually is.

Our structural data for hGWL-KinDom confirms the presence of both tail / linker and hydrophobic motif binding pockets (HM-pocket) in the N-terminal lobe of the minimal kinase domain, as originally proposed by the laboratories of Goldberg [18] and Castro [24]. It also reveals the precise molecular details for the interactions made by the amino acids of the C-lobe anchor (CLA) and tether (CLT) regions, back to the main body of the kinase domain (Figure 2C).

As previously indicated, whilst the C-terminal extension of GWL is of sufficient length for the cluster of phosphorylation sites at the AST (pThr873, pSer875, and pSer878) to potentially interact with the tail / linker binding site of the N-lobe, it is of an insufficient length for any direct interaction with the HM-pocket [18, 24]. Which other cellular proteins interact with the HM-pocket of GWL remains an important open and unanswered question in the field. Vigneron *et al*., have proposed that other AGC kinases, which contain a HM, directly interact with GWL; however, the identity of such kinases remains unknown. A further interesting possibility is that residues within the NCMR may fulfil this role, serving to stabilise and activate the kinase activity of GWL, without the need for additional protein partners. Clearly, more experimental work is required to address these particular points.

Until now, there has been a paucity in pharmacologic probes of GWL function and cellular biology. We describe herein the compound GKI-1, a novel ‘first generation inhibitor’ of human GWL that shows activity in cells against its intended target as judged by the observed reduction in ENSA phosphorylation levels. Despite the *in vitro* off-target range of GKI-1, it appears to cause cellular phenotypes that are surprisingly similar to the effects caused by GWL siRNA treatment (i.e. delayed mitotic entry and aberrant mitotic progression) [1, 15]. However, GWL depletion causes a more pronounced failure in cytokinesis that we only rarely observed in GKI-1 treated cells. This difference could be due to the incomplete inactivation of the kinase by the compound, or could point to a kinase-independent function of GWL. Cleary, further work will be required to precisely define on- and off-target effects of GKI-1 and to improve its potency and specificity by developing 2^nd^ generation inhibitors based on this compound.

## EXPERIMENTAL PROCEDURES

Full experimental procedures are provided in Supporting Information.

### Expression constructs

#### hGWL^FL^

The mammalian expression construct for N-terminally FLAG-tagged full-length human GWL has previously been described [28].

#### hGWL-KinDom

A synthetic gene was purchased, codon-optimised for expression in *E. coli* - that encoded amino acids (aa) 1-194 + ‘RTFC’ + 740-879 of hGWL; where as indicated, the ‘RTFC’-linking sequence was taken from PKCα. The gene was subsequently sub-cloned into the expression vector pTHREE-E, which adds a rhinovirus 3C-protease cleavable GST affinity-tag to the front of the encoded protein.

### Expression and purification of hGWL-KinDom

Expression and purification of hGWL-KinDom was carried out using standard chromatographic methods, using Amintra Glutathione Resin [Expedeon] and a HiLoad 26/60 Superdex 75 size exclusion chromatography column [GE Healthcare].

### Crystallisation

Crystallisation, data collection, phasing and refinement were performed as described in Supporting Information. Statistics for data collection and model refinement are provided in Supporting Information Table S1.

### Thermal shift assay

Assays were performed using a modified version of the protocol previously described in [41].

### Antibodies and western analysis

Polyclonal rabbit anti-GWL antibodies were generated and purified by Eurogentec. Mouse anti-Flag M2, rabbit [Prestige Antibodies] and mouse (RIPLY 74C) anti-MASTL (GWL) antibodies were purchased from Sigma-Aldrich and Abcam respectively. Polyclonal rabbit anti-phospho(Ser67)-ENSA/ARPP19 antibody was generated by Generon. Secondary antibodies were HRP-conjugated, polyclonal goat-derived antibodies against mouse and rabbit [Dako, Agilent Technologies].

### Kinase assays

Full methods are provided in Supporting Information.

### Inhibitor screens with hGWL-KinDom

Two small-molecule compound libraries, enriched with pharmacophores known to interact with the ATP-binding site of protein kinases, were screened as a pooled library in a Kinase-GloMax luminescence-kinase assay.

### Synthesis of GKI-1/2

All commercial reagents were purchased from Sigma-Aldrich, Alfa Aesar, Apollo Scientific, or Fluorochem and of the highest available purity. Anhydrous solvents were purchased from Acros (AcroSeal) or Sigma-Aldrich (SureSeal) and were stored under nitrogen. Proton nuclear magnetic resonance spectra were recorded at 500 MHz on a Varian VNMRS 500 MHz spectrometer, at 30°C. Carbon Nuclear Magnetic Resonance spectra were recorded at 125 MHz on a Varian 500 MHz spectrometer. LCMS data were recorded on a Waters 2695 HPLC using a Waters 2487 UV detector and a Thermo LCQ ESI-MS. Samples were eluted through a Phenomenex Lunar 3μ C18 50 mm × 4.6 mm column, using water and acetonitrile acidified with 0.1% formic acid at 1 ml/min and detected at 254 nm. The gradient employed was a 10 min. method of 5-95% MeCN. Mass Spectra (HRMS) were recorded at the University of Sussex Mass Spectrometry Centre on a high-resolution Orbitrap-XL instrument (Thermofisher). All experiments were carried out under an inert atmosphere of N_2_ unless otherwise stated.

### Synthesis of 4-(3-bromophenyl)-1-(oxan-2-yl)-1H-pyrazole (1A)

A solution of 4-(3-bromophenyl)-1H-pyrazole (1000 mg, 4.48 mmol) in toluene (30 mL) was treated with 3,4-dihydro-2H-pyran (0.49 mL, 5.38 mmol) and a few drops of trifluoroacetic acid (0.03 mL, 0.45mmol). The reaction mixture was heated to 100°C for 6 hours then cooled to room temperature and washed with saturated sodium bicarbonate solution, dried (MgSO_4_) and concentrated to dryness under reduced pressure. The crude was purified by flash chromatography eluting with a gradient of petroleum ether: EtOAc (0 to 30% EtOAc). The desired fractions were evaporated to dryness *in vacuo* to afford the product as a white solid (1370 mg, 98% yield). ^1^H NMR (500 MHz, DMSO-d6) δ 8.43 (s, 1H), 7.99 (s, 1H), 7.84 (s, 1H), 7.61 (d, J = 7.8 Hz, 1H), 7.36 (d, J = 7.8 Hz, 1H), 7.29 (t, J = 7.8 Hz, 1H), 5.38 (dd, J = 10.0, 2.3 Hz, 1H), 3.96 - 3.85 (m, 1H), 3.68 - 3.55 (m, 1H), 2.16 - 2.02 (m, 1H), 1.99 - 1.88 (m, 2H), 1.75 - 1.60 (m, 1H), 1.57 - 1.46 (m, 2H).

### Synthesis of N-(4-chlorophenyl)-3-[1-(oxan-2-yl)-1H-pyrazol-4-yl]aniline (THP-GKI-1)

A mixture of 4-(3-bromophenyl)-1-(oxan-2-yl)-1H-pyrazole (1300 mg, 4.23 mmol), 4-chloroaniline (0.57 mL, 5.08 mmol), tris(dibenzylideneacetone)dipalladium(0) (78.67 mg, 0.09 mmol), XPhos (161.2 mg, 0.34 mmol) and sodium tert-butoxide (569.39 mg, 5.92 mmol) in toluene (25 mL) was purged with nitrogen and heated to 100°C for 3 hours. The reaction mixture was cooled to room temperature and washed with water and brine, dried (MgSO_4_) and concentrated to dryness under reduced pressure. The crude was purified by flash column chromatography eluting with a gradient of petroleum ether: EtOAc (0 to 30%). The desired fractions were concentrated to dryness *in vacuo* to afford the product as a white solid (1459 mg, 92% yield). ^1^H NMR (500 MHz, DMSO-d6) δ 8.26 (s, 1H), 8.24 (s, 1H), 7.83 (s, 1H), 7.28 - 7.17 (m, 4H), 7.12 - 7.01 (m, 3H), 6.89 (d, J = 8.1 Hz, 1H), 5.38 (dd, J = 10.0, 2.3 Hz, 1H), 3.96 - 3.88 (m, 1H), 3.66 - 3.54 (m,1H), 2.17 - 2.02 (m, 1H), 1.98 - 1.84 (m, 2H), 1.74 - 1.59 (m, 1H), 1.57 - 1.46 (m, 2H).

### Synthesis of N-(4-chlorophenyl)-3-(1H-pyrazol-4-yl)aniline (GKI-1)

A solution of N-(4-chlorophenyl)-3-[1-(oxan-2-yl)-1H-pyrazol-4-yl]aniline (1550 mg, 4.38 mmol) in methyl alcohol (30 mL) was treated with concentrated hydrochloric acid (1.99 mL, 21.9 mmol) and the reaction mixture stirred at room temperature for 3 hours. The reaction mixture was neutralised with aqueous 1M NaOH solution and the precipitate filtered under suction and washed with water. The solid was then re-dissolved in EtOAc, washed with brine, dried (MgSO_4_) and solvent evaporated to dryness under reduced pressure. The crude was triturated with hot dichloromethane and dried under high vacuum to afford the product as a white solid (625 mg, 52% yield). ^1^H NMR (500 MHz, DMSO-d6) δ 12.89 (s, 1H), 8.26 (s, 1H), 8.09 (s, 1H), 7.82 (s, 1H), 7.29 - 7.16 (m, 4H), 7.13 - 6.99 (m, 4H), 6.89 (d, J = 7.9 Hz, 1H); ^13^C NMR (125Mhz, DMSO-d6) δ 143.7, 143.2, 134.4, 130.1, 129.0, 125.8, 123, 121.8, 118.2, 115.6, 114.8; LCMS-LCQ: 10 mins, 5-95% MeCN, Rt = 6.63, > 99% purity; [M+H]^+^ 270.20; HRMS (ESI) calculated for C_15_H_12_ClN_3_ (M + H+) 270.0790, found 270.0793.

### Synthesis of 4-(4-bromophenyl)-1-(oxan-2-yl)-1H pyrazole (1B)

A solution of 4-(4-bromophenyl)-1H-pyrazole (2150 mg, 9.64 mmol) in toluene (50 mL) was treated with 3,4-dihydro-2H-pyran (0.88 mL, 9.64 mmol) and trifluoroacetic acid (0.07 mL, 0.96 mmol). The reaction mixture was heated to 100°C for 6 hours then cooled to room temperature and washed with saturated sodium bicarbonate solution, dried (MgSO_4_) and concentrated to dryness under reduced pressure. The crude was purified by flash chromatography eluting with a petroleum ether:EtOAc gradient (0 to 30% EtOAc). The desired fractions were concentrated to dryness *in vacuo* to afford the product as a white solid (2.97 g, 96% yield). ^1^H NMR (500 MHz, DMSO-d6) δ 8.37 (s, 1H), 7.94 (s, 1H), 7.57 (d, J = 8.6 Hz, 2H), 7.52 (d, J = 8.6 Hz, 2H), 5.38 (dd, J = 9.9, 2.4 Hz, 1H), 3.96 - 3.87 (m, 1H), 3.67- 3.56 (m, 1H), 2.15 - 2.02 (m, 1H), 1.99 -1.86 (m, 2H), 1.74 - 1.59 (m, 1H), 1.57 - 1.47 (m, 2H).

### Synthesis of N-(4-chlorophenyl)-4-[1-(oxan-2-yl)-1H-pyrazol-4-yl]aniline (THP-GKI-2)

A mixture of 4-(4-bromophenyl)-1-(oxan-2-yl)-1H pyrazole (500 mg, 1.63 mmol), 4-chloroaniline (0.22 mL, 1.95 mmol), tris(dibenzylideneacetone)dipalladium(0) (30.26 mg, 0.0300 mmol), XPhos (62 mg, 0.13 mmol) and sodium tert-butoxide (219 mg, 2.28 mmol) in toluene (10 mL) was purged with nitrogen and heated to 100°C for 3 hours. The reaction mixture was cooled to room temperature and washed with water and brine. The organic phase was dried (MgSO_4_) and concentrated to dryness under reduced pressure. The crude was purified by flash column chromatography eluting with a gradient of petroleum ether:EtOAc (0 to 30%). The desired fractions were evaporated *in vacuo* to afford the product as a white solid (344 mg, 58% yield). ^1^H NMR (500 MHz, DMSO-d6) δ 8.27 (s, 1H), 8.18 (s, 1H), 7.82 (s, 1H), 7.47 (d, J = 8.6 Hz, 2H), 7.22 (d, J = 8.9 Hz, 2H), 7.07 - 6.97 (m, 4H), 5.37 (dd, J = 10.0, 2.3 Hz, 1H), 3.96 - 3.87 (m, 1H), 3.68 - 3.57 (m,1H), 2.09 (d, J = 7.6 Hz, 1H), 1.96 - 1.86 (m, 2H), 1.75 - 1.60 (m, 1H), 1.59 - 1.47 (m, 2H).

### Synthesis of N-(4-chorophenyl)-4-(1H-pyrazol-4-yl)aniline (GKI-2)

A solution of N-(4-chorophenyl)-4-[1-(oxan-2-yl)-1H-pyrazol-4-yl]aniline (344 mg, 0.97 mmol) in ethanol (10 mL) was treated with concentrated hydrochloric acid (0.44 mL, 4.86 mmol) and the reaction mixture stirred for 3 hours at room temperature then cooled to room temperature and neutralised with 1 M aqueous sodium hydroxide solution. A precipitate was collected by filtration, washed with water and dried under suction. The solid was re-crystallised from hot acetonitrile to afford the product as a white solid (106 mg, 39% yield). ^1^H NMR (500 MHz, DMSO-d6) δ 12.80 (s, 1H), 8.23 (s, 1H), 8.03 (s, 1H), 7.80 (s, 1H), 7.46 (d, J = 8.5 Hz, 2H), 7.21 (d, J = 8.8 Hz, 2H), 7.09 - 6.94 (m, 4H); ^13^C NMR (125Mhz, DMSO-d6) δ 143.3, 141.2, 136.2, 129.4, 126.5, 125.9, 14.9, 122.7, 121.7, 118.5, 117.9; LCMS-LCQ: 10 mins, 5-95% MeCN, Rt = 6.51 mins, > 99% purity; [M+H]^+^ 270.16; HRMS (ESI) calculated for C_15_H_12_ClN_3_ (M + H+) 270.0790, found 270.0794.

### Molecular docking - AutoDock

GKI-1/2 were docked into the ATP-binding site of the hGWL-KinDom crystal structure using AutoDock 4.2.6. Full methods are provided in Supporting Information.

### Cell culture

HeLa cells were cultured in Dulbecco's modified Eagle Medium (DMEM) supplemented with 10% v/v FBS, 2 mM L-glutamine, 100 U/ml penicillin and 0.1 mg/ml streptomycin in a 37°C, 5% CO_2_ incubator.

### Immunofluorescence and Time-lapse video microscopy

HeLa cells were plated on glass coverslips and then transfected with siRNAs using Lipofectamine RNAiMax [ThermoFisher Scientific]. 48 hours after treatment, cells to be used for immunofluorescence were treated with nocodozole and DMSO, or GKI-1. After fixing and staining with antibodies, cells were visualised with a ScanR High Content Screening Station [Olympus Life Science]. Time-lapse videos for treated cells were recorded on an Olympus IX3 microscope fitted with an Ocra-Flash 4.0 CMOS camera [Hamatsu].

## SUPPORTING INFORMATION










