# Supplementary material for: A first generation inhibitor of human Greatwall kinase, enabled by structural and functional characterisation of a minimal kinase domain construct
Source: Oncotarget. 2016 Aug 22;7(44):71182–97. doi: 10.18632/oncotarget.11511 (PMC5342071; doi:10.18632/oncotarget.11511)
Supplement: Supplementary file 1 [file oncotarget-07-71182-s001.pdf]

SUPPORTING INFORMATION

A first generation inhibitor of human Greatwall kinase, enabled by structural and functional characterisation of a minimal kinase domain construct

TABLE OF CONTENTS **Page**

Materials and Methods S2-S4

Figure S1. Purification of hGWL-KinDom S5

Figure S2. Detection of phosphorylation sites in hGWL-KinDom produced in *E. coli* S6

Figure S3. Representative region of $2mF_o$-DF_c electron density map S7

Figure S4. Inhibition of immunoprecipitated $hGWL^{FL}$ S8

Figure S5. Screening of pooled small-molecule compound library S9

Figure S6. Confirmatory screen of GR1-1-4 using immunoprecipitated $hGWL^{FL}$ S10

Figure S7. Inhibition of hGWL-KinDom by GRI-3 and GRI-4 S11

Figure S8. Molecular Docking of GKI-1 and GKI-2 / Inhibition of $hGWL^{FL}$ by GKI-2 S12

Figure S9. Kinase phylogeny vs GKI-1 inhibitory activity S13

Table S1. Crystallography: Data collection and refinement statistics S14

Table S2. Pairwise distance alignment search of GWL ligand-binding site S15

References S16

Supplementary Videos — HeLa cells treated with:

SV1: scrambled siRNA control; **SV2:** 25 µM GKI-1, **SV4:** 50 µM GKI-1, **SV4:** siGWL

Materials and Methods

Expression and purification of hGWL-KinDom

A codon-optimised synthetic gene encoding hGWL-KinDom was cloned into the expression vector pTHREE-E, which adds a rhinovirus 3C-protease cleavable GST affinity-tag to the front of the encoded protein. *E. coli* strain Rosetta2(DE3) was transformed with pTHREE-E/hGWL-KinDom [Merck-Millipore, Watford, UK]. A 'starter culture' was prepared by inoculating 50 ml of Turbo-broth [Molecular Dimensions, Newmarket, UK] supplemented with 100 μg/ml ampicillin and 34 μg/ml chloramphenicol. This was then incubated at 37 °C, in an orbital shaking incubator set at 220 rpm, until the absorbance at a wavelength of 600nm (A600) reached 1.0. The culture was then stored overnight at 4 °C. The following day, 10 ml of the starter culture was used to inoculate 1 litre of Turbo-Broth in a 2 litre Erlenmeyer flask (supplemented as before with antibiotics). Again, the culture was incubated at 37 °C, in an orbital shaking incubator set at 220 rpm, until the A600 reached 2.0. The Erlenmeyer flask was then placed on ice and the temperature of the incubator reduced to 16 °C. When this temperature was attained, the flask was placed back into the incubator, and recombinant protein expression induced by the addition of IPTG (isopropyl β-D-1-thiogalactopyranoside) to a final concentration of 0.5 mM. The culture was then incubated for a further period of ~16 hours. Cells were then harvested by centrifugation (5000 x g, 10 minutes, 4 °C) and the resultant pellet stored at -20 °C until required.

The frozen cells were resuspended in 2 ml / gram of cell pellet of Buffer A: 50 mM HEPES.NaOH pH 7.5, 500 mM NaCl, 0.5 mM Tris(2-carboxyethyl)phosphine hydrochloride (TCEP) supplemented with protease inhibitor tablets [Roche Diagnostics, Burgess Hill, UK] and 30-40 μl of Benzonase [Merck Millipore]. Cells were disrupted by sonication, and the insoluble material and debris removed by centrifugation (40,000 x *g*, 30 minutes, 4 °C).

The supernatant arising from this step was applied to a batch / gravity column containing 10 ml of Amintra Glutathione Resin [Expedeon Ltd, Harston, UK] pre-equilibrated in Buffer A. The column containing the cell extract and resin was rotated / rolled at 4 °C for a period of 1-2 hours to facilitate protein binding, and then allowed to pack under gravity flow. The resin was then washed with the addition of 10 column volumes (CV) of Buffer A to remove any unbound material, then re-suspended in an equal volume of Buffer B: 50 mM HEPES.NaOH pH 7.5, 500 mM NaCl, 150 mM KCl, 10 mM $MgCl_2$, 10 mM ATP, 0.5 mM TCEP, and rotated / rolled for a further period of 2 hours at 4 °C. The resin was once again allowed to pack under gravity flow, followed by an additional wash with a further 5 CV of Buffer A. Rhinovirus 3C-protease was then added to the resin slurry (200 μl of a 2 mg/ml stock solution) and the cleavage reaction allowed to proceed overnight, with rotation / rolling at 4 °C as before.

The following day, the resin was again allowed to pack under gravity flow, but this time collecting the flow-through. Fractions containing hGWL-KinDom were identified by SDS-PAGE, pooled, and then concentrated using centrifugal ultra-filtration [10,000 MWCO Vivaspin 20, Sartorius Stedim UK Ltd, Epson, UK], before being applied to a HiLoad 26/60 Superdex 75 size exclusion chromatography column [GE Healthcare, Litte Chalfont, UK], pre-equilibrated in Buffer C: 20 mM HEPES.NaOH pH 7.5, 300 mM NaCl, 0.5 mM TCEP. Again, fractions containing hGWL-KinDom were identified by SDS-PAGE, pooled, and then concentrated to a final concentration of 12 mg/ml, and stored at -80 °C until required.

Crystallisation and data collection

hGWL-KinDom was co-crystallised with staurosporine (STU), by firstly mixing purified protein at 12 mg/ml with STU (20 mM stock solution) in a 1:3 protein:ligand ratio, and then incubating on ice for a period of 3 hours, before setting up sitting drop vapour diffusion experiments at 4 °C; where 1 μl of complex was mixed with 1 μl of crystallisation buffer and equilibrated against a well volume of 50μl. Crystals were obtained in condition D12 of the PEG/Ion HT screen (0.2 M ammonium citrate, 20% w/v polyethylene glycol 3350; Hampton Research, California, USA). Crystals were swiped successively through buffers containing increasing concentrations of cryo-protectant, before being plunged into liquid nitrogen; 30% v/v glycerol was sufficient to prevent ice formation. Diffraction data to 3.1 Å resolution were collected at the Diamond Light Source [DLS, Didcot, UK] on beamline I03.

Phasing, model building and refinement

All diffraction data were collected at 100K. Data were integrated using the software package XDS (1), and then processed using the Pointless / Aimless / Ctruncate pipeline of the CCP4 software suite (2-5). Phases were obtained by molecular replacement using Phaser (6) with a polyalanine model based on PDB: 1H1W (PDK1; (7)) as a search model. An iterative combination of manual building in Coot (8) and refinement with Phenix.refine (9) produced the final model.

The protein crystallised in spacegroup $P2_12_12_1$, with two molecules comprising the asymmetric unit. For one molecule (chain A), amino acids 30-72, 86-177 and 740-854 are readily discernible in electron density maps, whilst for the other (chain B) amino acids 30-72, 86-177 and 740-854 are readily discernible.

Thermal shift assay

For thermal denaturation, samples containing protein at 1.5 μM and 5 x SYPRO Orange (diluted from a 5000 x stock supplied in DMSO; Sigma-Aldrich, Gillingham, UK) were prepared in sample buffer: 20 mM HEPES.NaOH pH 7.5, 300 mM NaCl, 0.5 mM TCEP. Denaturation curves were monitored in 96-well PCR plates using a Roche LightCycler 480 II, with 465 and 580 nm filters for excitation and emission wavelengths, respectively. The program was as follows: 1 min at 20°C, followed by a continuous increment of 0.03°C/s to a final temperature of 85°C. Temperature midpoints (Tm) for each folded to unfolded transition were determined by non-linear regression fitting of a modified Boltzmann model to normalized data in Prism 6.0 (version 6.0h, GraphPad Software, La Jolla, CA USA).

$$Y = (a_n X + b_n) + \frac{(a_d X + b_d) - (a_n X + b_n)}{1 + e^{\frac{T_m - X}{m}}}$$

Where: a_n and a_d are the slopes, and b_n and b_d the y-intercepts, of the native and denatured baselines respectively. T_m is the temperature midpoint of the transition from native to denatured states, and m represents a generic slope factor.

Kinase Assays

Non-radioactive kinase assays were set-up by combining hGWL-KinDom (305 μM in 20 mM HEPES pH 7.5, 500 mM NaCl, and 0.5 mM TCEP) diluted to desired concentration or immunoprecipitated $hGWL^{FL}$ on beads with ultra-pure ATP [Promega, 25 μM] and His-tagged substrate α-endosulfine (ENSA, 2 μM) in kinase assay buffer (50 mM Tris-HCl pH 7.5, 0.5 mM EGTA, 150 mM NaCl, 0.1% v/v NP-40 and 15 mM $MgCl_2$). The reaction was allowed to proceed for 30 min at 37 °C or another indicated time-point after which it was stopped with either SDS loading buffer (5X buffer, including 0.25 % w/v Bromophenol blue, 0.5 M DTT (dithiothreitol), 50% v/v Glycerol, 10% v/v SDS (sodium dodecyl sulphate), and 0.25M Tris-HCl pH 6.8) or Kinase-Glo Max reagent [Promega]. Recombinant His-tagged ENSA was purified using Ni-NTA agarose [QIAGEN] from BL21 *E. coli* following the manufacturer's recommended protocol.

Radioactive Kinase Assay

To detect ENSA phosphorylated at Ser67 (p-ENSA), the standard kinase reaction was spiked with 0.075 MBq γ-^{32}P ATP (PerkinElmer, Seer Green, UK) per 20 μl reaction. After the reaction was stopped with 5 μl 5X SDS loading buffer and boiled for 5 min at 95 °C the mixture was resolved via SDS-PAGE (4-15% Criterion pre-cast gels; Bio-Rad Laboratories, Hemel Hempstead, UK, or 13% SDS-polyacrylamide gels). Staining with coomassie-blue revealed an ~17 kDa band that was imaged by autoradiography. All kinase assays were repeated thrice per assay condition and each point on the concentration-dependent response curves represents the mean ± 1 S.D.

Kinase-Glo Max Assay

After stopping the above non-radioactive kinase assay with 1 volume of Kinase-Glo Max reagent the mixture was allowed to incubate for a further 15 min. To detect consumption of ATP, samples were transferred to white, flat-bottom 96-well plates [Thermo Fisher Scientific, Loughborough, UK] and luminescence was detected using a POLARstar Omega micro-plate reader [BMG Labtech GmbH, Ortenberg, Germany]. As this assay measures ATP, activity or ATP turnover was measured as the -ΔRLU. Non-linear regression using Prism 6.0 and the built-in enzyme kinetics module were used to determine Michaelis-Menten kinetic parameters, k_{cat} and K_m, for both ATP and ENSA. All kinases assays were conducted thrice in triplicate (n = 9) and IC_{50} values are reported as the mean ± 1 S.D.

Immunoprecipitation Kinase Assay

HEK293T cells were transfected with 10 μg of plasmid (Flag-tagged $hGWL^{FL}$ or GFP; pcDNA3-EGFP, Addgene, Cambridge MA, USA) using $CaCl_2$. 48 hours later 100 ng/ml nocodazole was added to the media and incubated for a further 16–18 h. Cells were collected and washed in PBS-A followed by lysis on ice for 20 min in 1mL IP buffer (50 mM Tris-HCl pH 7.5, 150 mM NaCl, 10% v/v glycerol, 0.5% v/v NP-40, 1 mM EDTA supplemented with protease and phosphatase inhibitors (Complete and PhosStop; Roche Diagnostics)). Samples were then sonicated and clarified by centrifugation at 13,000 rpm for 30 min at 4°C. To capture Flag-tagged

hGWL^{FL}, 100 μl of lysate was diluted 5-fold with IP buffer and combined with 3 μl anti-Flag M2 magnetic Dynabeads [Sigma-Aldrich] overnight at 4 °C. After sequential washes with IP buffer, 1:1 mix of IP and kinase assay buffers and a final wash in kinase assay buffer, the beads were then used in the kinase assay. The reaction was stopped by adding SDS loading buffer and analysed by western blot: p-ENSA and GWL levels were detected using the anti-phospho(Ser67)-ENSA and anti-MASTL(GWL, Prestige) antibodies. Activity was measured as the p-ENSA/GWL ratio and % kinase activity was normalised to the DMSO control. All kinases assays were repeated thrice per assay condition and each point on the concentration-dependent response curves represents the mean ± 1 S.D.

PKA, ROCK1 and CDK2 kinase assays
PKA and ROCK1 assays (Promega) were carried out according to the manufacturer's protocols and CDK2 assays were carried out with Histone H1 as substrate (1 μg per 20 μl reaction) according to our Kinase-Glo Max assay. CDK2 was expressed and purified from *E. coli* as previously described (10).

Inhibitor Screens with hGWL-KinDom
Two small-molecule compound libraries, enriched with pharmacophores known to interact with the ATP-binding site of protein kinases, were kindly provided by GlaxoSmithKline and Roche. We used this pooled library (582 compounds, 10 μM screening concentrations) in a Kinase-Glo Max luminescence kinase assay (11, 12) as part of a two-point screen against hGWL-KinDom kinase activity, using ATP at fixed concentrations of 15 μM and 45 μM; ~1- and 3-fold the Km value for ATP respectively. With STU as positive control at a concentration of 200 μM, Z-factor values for screening plates ranged from 0.41 to 0.69 (**Supplementary Figure 4A,B**). A threshold value of 3σ was used to identify 34 hits, all with inhibitory activity greater than 30% (hit rate = 5.8%; **Supplementary Figure 4A**).

Molecular Docking - AutoDock
AT13148 and GKI-1/2 were docked into the ATP-binding site of the hGWL-KinDom crystal structure (where the disordered C-helix was homology-modelled) using AutoDock 4.2.6. PDB format files for the ligand and kinase domain were pre-processed using AutoDock Tools 1.5.6 as described in Mohamed, *et al.* (13). (**Figure 4 and Supplementary Figure 6A,B**).

Immunofluorescence and Time-lapse video microscopy
2.5 X 10^5 HeLa cells were plated onto glass coverslips in a 6-well plate, or 4-well μ-slides [Ibidi GmbH] were seeded with 5 X 10^4 cells and then transfected with Qiagen AllStars negative control or Hs_MASTL_6 siRNAs (14) using Lipofectamine RNAiMax [ThermoFisher Scientific]. Media was exchanged with fresh media the next day. 48 hours after siRNA treatment, only cells to be used for immunofluorescence (IF) were treated with nocodazole (200 ng/mL) and DMSO or GKI-1 at the indicated concentration. For IF, cells were fixed, stained with primary mouse anti-GWL and rabbit anti-p-ENSA antibodies, secondary Alexa Fluor 647-conjugated anti-rabbit and Alexa Fluor 488-conjugated anti-mouse antibodies, DAPI and mounted onto glass microscope slides using Diamond Antifade Mountant [Molecular Probes, ThermoFisher Scientific]. Quantitation of IF images was performed using the ScanR High-Content Screening Station [Olympus Life Science] which acquired 220, 40x images per cover slip. Data were normalised against the AllStars negative control siRNA treated cells and values obtained from three biological replicates were reported as the mean ± 1 S.D. To assist with selecting mitotic cells, ScanR imaging software was utilised to gate for mitotic cells using the circularity factor and total DAPI parameters.

To produce time-lapse videos, after 48 hours and cell treatment with DMSO or compound (cells were treated with GKI-1 for 4-h before image acquisition) cells plated in 4-well μ-slides were imaged every 5 minutes for 8.5 hours on an Olympus IX73 microscope, using a 40 x 0.95NA air objective, and recorded on an Orca-Flash 4.0 CMOS camera [Hamatsu]. The computer program Fiji (http://www.fiji.sc/Fiji) was used to generate videos from image sequence TIF files. Data were normalised against the AllStars negative control siRNA treated cells and values obtained from 3 – 5 biological replicates were reported as the mean ± 1 S.D.

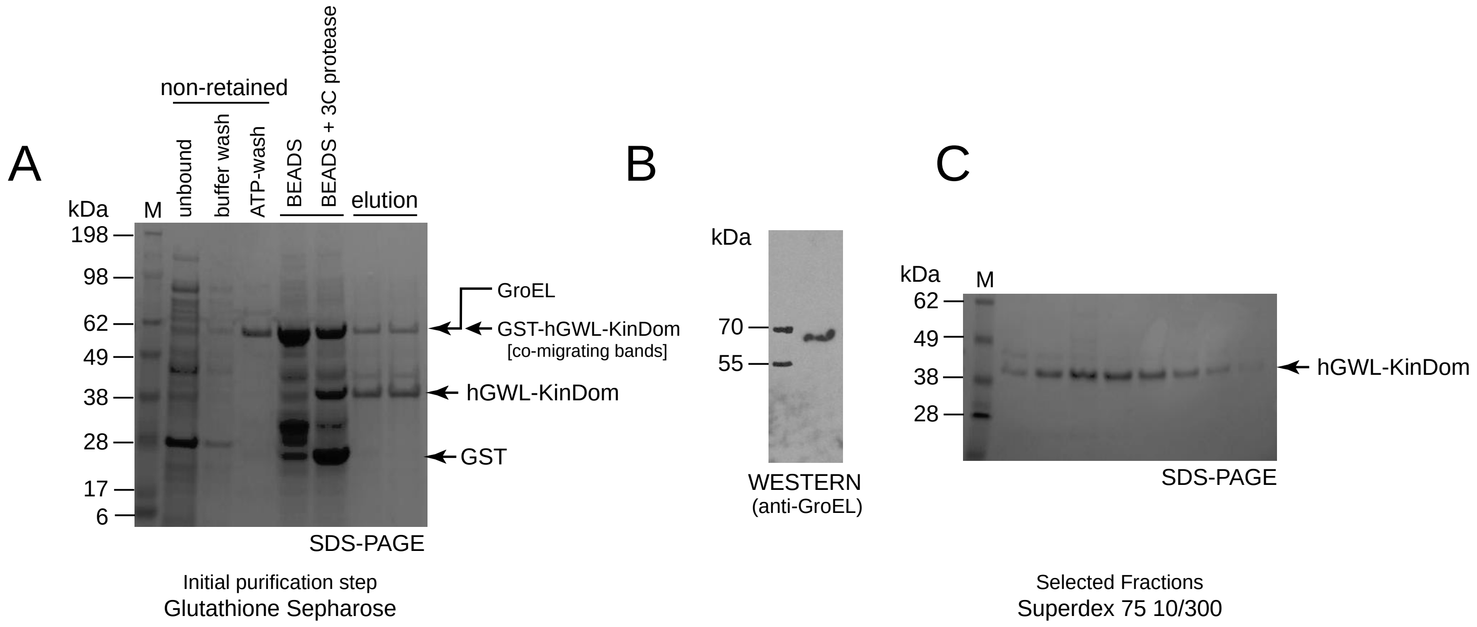

Figure S1. Purification of hGWL-KinDom

(A) SDS-PAGE gel of selected fractions, collected during purification of hGWL-KinDom. M = molecular mass ladder. The hGWL-KinDom GST-fusion migrates at the same position as GroEL. **(B)** Anti- GroEL western blot (ab90522, Abcam, Cambridge UK). **(C)** SDS-PAGE gel of fractions from the final size-exclusion chromatography step (HiLoad 26/60 Superdex 75, GE Healthcare, Little Chalfont, UK).

A

		Relative Amount	P-value (PEP)	Score
T17	K E P G G G A A T*(97.6) E E G V N R	$1.8X10^{-4}$	$1.5X10^{-4}$	97
T868	N T*(45.5) A Q H L T(-45.5) V S(-83.9) G F S(-141.8) L	0.16	$1.7X10^{-118}$	201
T873	N T(-35.63) A Q H L T*(30.6) V S(-30.6) G F S(-107.1) L	1	$6.2X10^{-24}$	149
S875	N T(-121.7) A Q H L T(-62.2) V S*(62.2) G F S(-76.7) L	0.37	$2.1X10^{-55}$	170
S878	N T A Q H L T V S G F S* L	<$1.8X10^{-4}$	$1.3X10^{-3}$	41

B

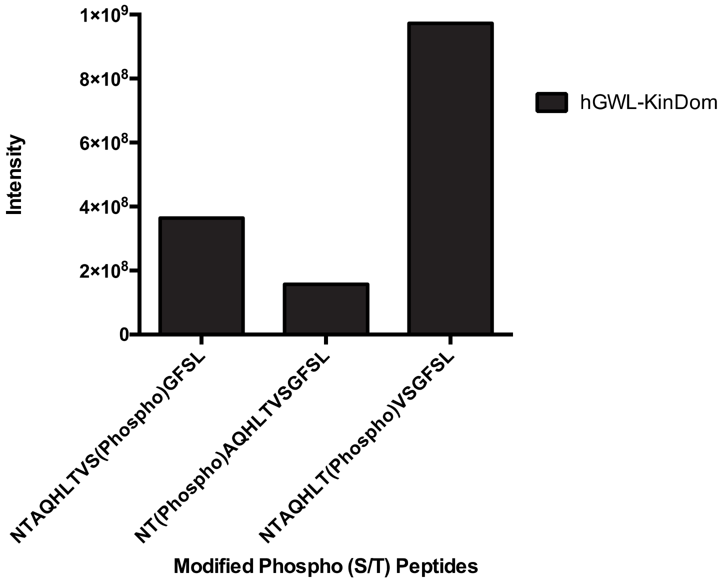

Figure S2. **Detection of phosphorylation sites in hGWL-KinDom produced in *E. coli***
(A) A band corresponding to hGWL-KinDom was subjected to in-gel digestion with trypsin before mass spectrometric (MS) analysis (Proteomics Facility, University of Bristol). All the raw spectra from MS were analyzed using MASCOT and MaxQuant as search engines, and searched against an in-house database containing either the hGWL-KinDom sequence or the UNIPROT_ALL.fasta database. The search parameters are as follows: Carbamidomethyl (C) as Fixed modification; Oxidation (M), Deamidated (NQ), Phospho (ST), Phospho (Y) as Variable modification, taxonomy selected to *Homo sapiens*, Peptide mass tolerance of ±10 ppm, fragment mass tolerance of 0.7 Da, allowing missed cleavage site of 1. Peptide identification was done using parameters such as peptide ion scores >31, which indicate identity or extensive homology ($p < 0.05$). Phospho-site Ser878 was identified using MASCOT and all others were identified using both MASCOT and MaxQuant.
(B) Quantitation of the most abundant phospho-peptides using MaxQuant.

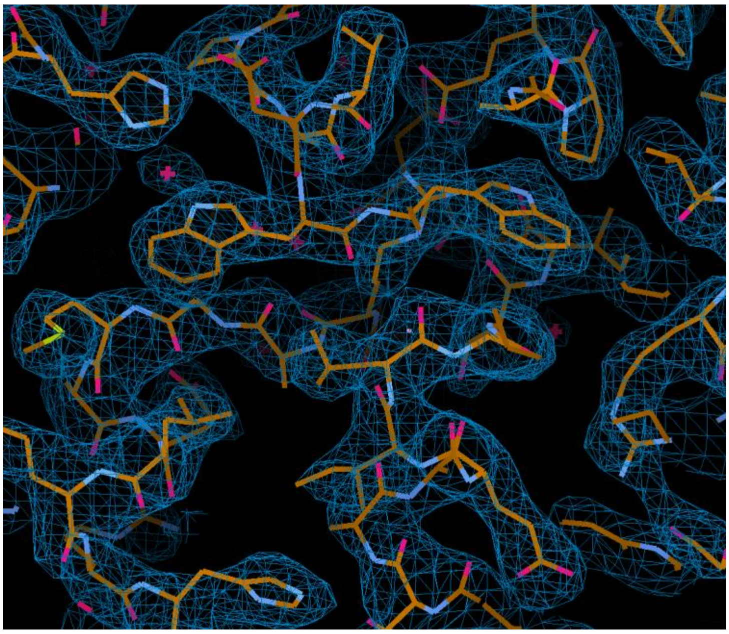

Figure S3. **Representative portion of $2mF_o$-DF_c electron density map**
Electron density is represented by the 'chicken wire mesh' coloured blue, and contoured at 0.26 e/Å^3, or 1.22 rmsd. The polypeptide chain of hGWL-KinDom is shown in 'stick' representation, with carbon atoms coloured orange. Nitrogen, oxygen and sulphur atoms are coloured cyan, red and yellow respectively.

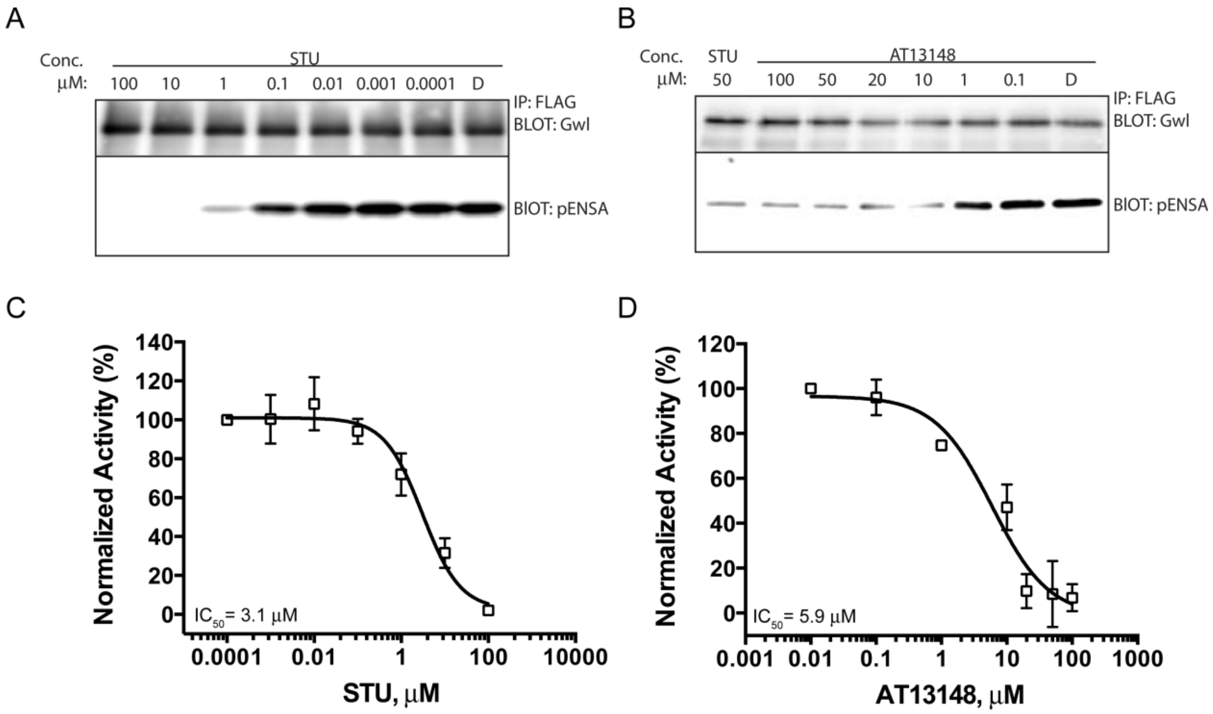

Figure S4. Inhibition of immunoprecipitated hGWL^{FL}

Immunoprecipitated Flag-tagged hGWL^{FL} was treated with increasing concentrations of **(A,C)** STU and **(B,D)** AT13148 and kinase activity was measured using the immunoprecipitation kinase assay. Non-linear regression with Prism 6.0 was used to determine IC_{50} values.

A

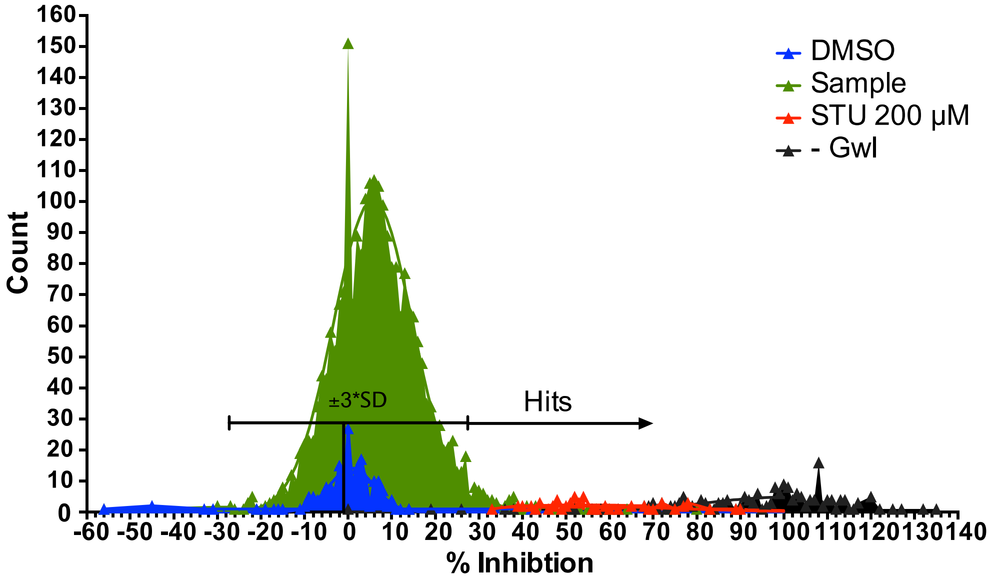

B

Plate	Run	ATP (μM)	Z-Factor
1	1	45	0.41
2	1	45	0.46
3	1	15	0.54
4	1	15	0.54
5	2	45	0.54
6	2	45	0.43
7	2	15	0.48
8	2	15	0.60

Figure S5. Screening of a pooled small-molecule compound library
(A) Two small-molecule compound libraries containing a total of 582 compounds were obtained from GlaxoSmithKline (GSK) and Roche. A 2-fold single-point screen of these libraries (10 μM screening concentration per inhibitor) using ATP concentrations of 15 and 45 μM and hGWL-KinDom were performed under Kinase-Glo Max assay conditions. The pan-kinase inhibitor staurosporine (STU, 200 μM) and reactions lacking enzyme were used as a positive control and DMSO as vehicle; Z-factor values for all the runs ranged from 0.41 – 0.69 **(B).** To identify hits, a threshold of 3 standard deviations of the DMSO controls was set, which led to identification of 34 'hits', all with an inhibitory activity greater than 30%; hit rate of 5.8%.

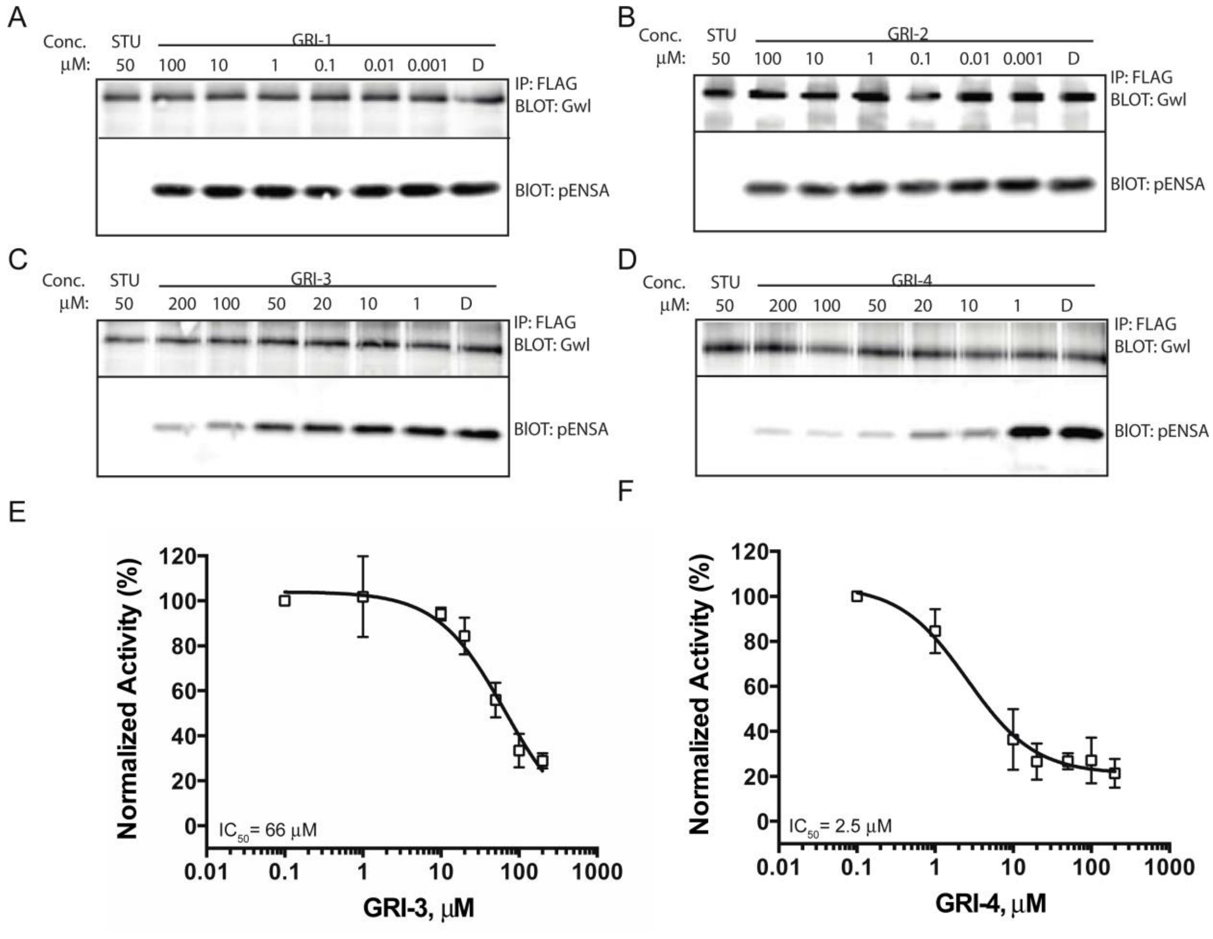

Figure S6. Confirmatory screen of GR1-1-4 using immunoprecipitated hGWL^{FL}
Immunoprecipitated Flag-tagged hGWL^{FL} was treated with increasing concentrations of **(A)** GRI-1, **(B)** GRI-2, **(C,E)** GRI-3 and **(D,F)** GRI-4 and kinase activity was measured using the immunoprecipitation kinase assay. Non-linear regression with Prism 6.0 was used to determine IC_{50} values.

A

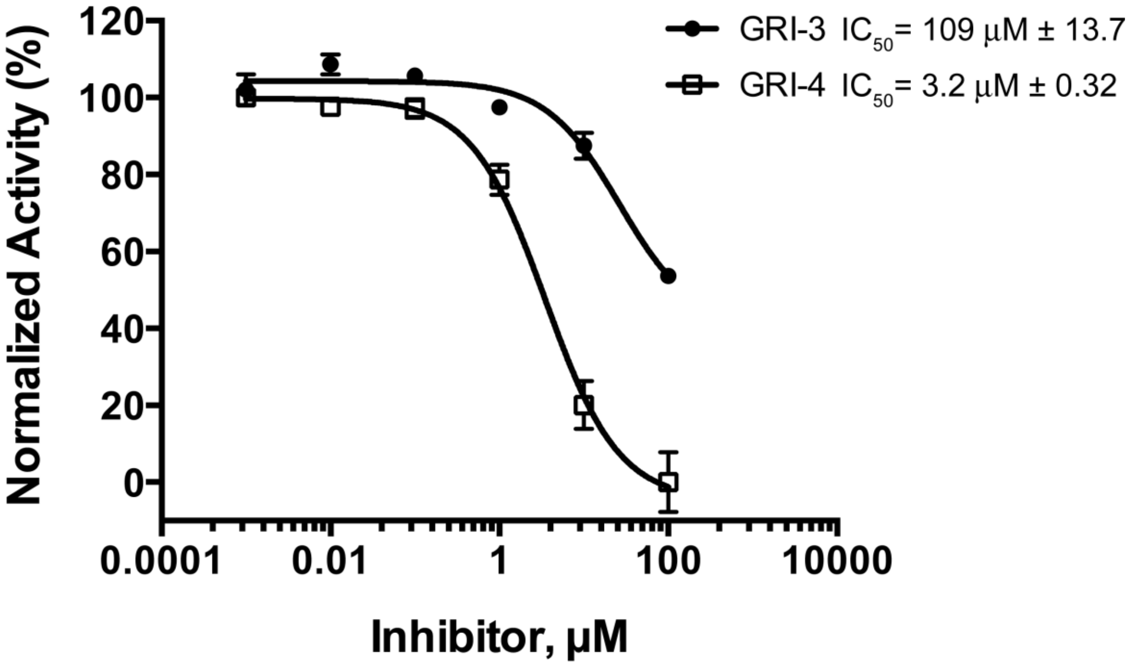

Figure S7. Inhibition of hGWL-KinDom by GRI-3 and GRI-4
hGWL-KinDom was treated with increasing concentrations of GRI-3and GRI-4 and kinase activity was measured using the Kinase-Glo Max assay. Non-linear regression with Prism 6.0 was used to determine IC_{50} values.

A

Binding Energy (kcal/mol)

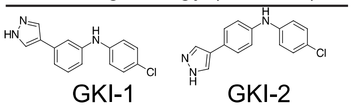

Conformations	GKI-1 (*m*-pyrazole)	GKI-2 (*p*-pyrazole)
1	-7.89	-7.43
2	-7.40	-7.46
3	-7.43	-7.46
4	-7.87	-7.45
5	-7.83	-7.45
6	-7.85	-7.45
7	-7.69	-7.46
8	-7.90*	-7.47
9	-7.86	-7.45
10	-7.88	-7.46

B

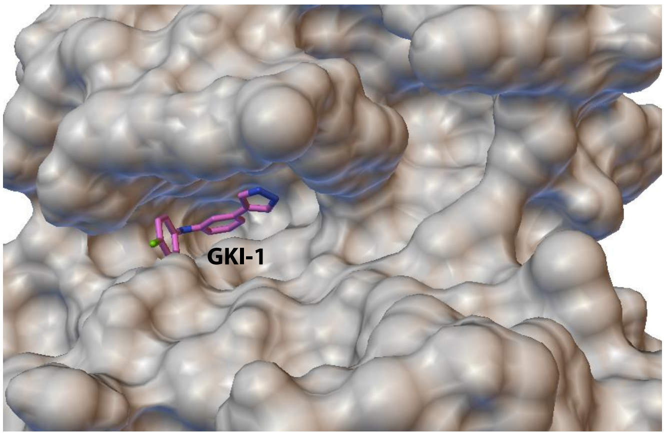

C

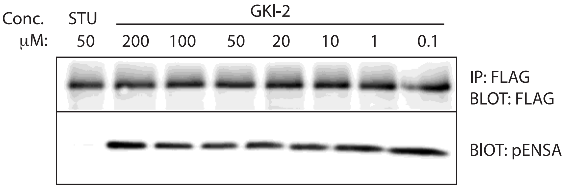

D

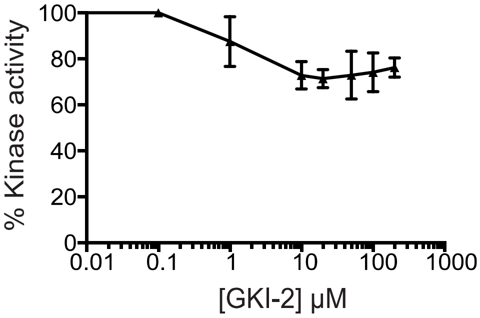

Figure S8. Inhibition of hGWL^{FL} by GKI-2 / Molecular Docking of GKI-1 and GKI-2
(A,B) Using Autodock 4.2.6 and AutoDock Tools 1.5.6, GKI-1 (*m*-pyrazole) and GKI-2 (*p*- pyrazole) were docked to the nucleotide-binding site of hGWL-KinDom. Each docking run produced 10 different poses. **(B)** GKI-1 taken from the 8th pose, modelled into the ATP-binding pocket. **(C,D)** Immunoprecipitated hGWL^{FL} was treated with increasing concentrations of GKI-2 and kinase activity was measured using the immunoprecipitation kinase assay. **(D)** % kinase activity was normalised to the DMSO control and plotted against GKI-2 concentration using Prism 6.0.

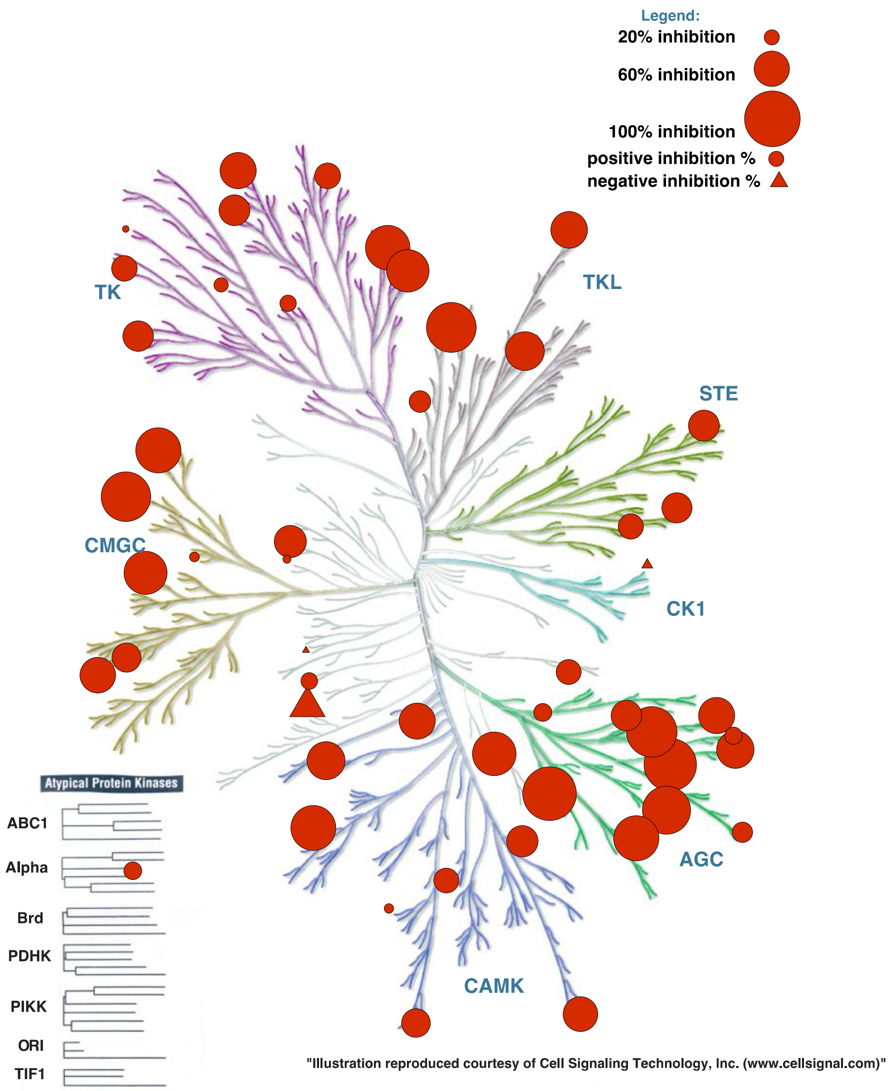

Figure S9. Kinase phylogeny vs GKI-1 inhibitory activity

Kinome render [Manning et al. (2002)] was used to generate a phylogenetic map with overlapping 25 μM GKI-1 inhibitory activity plotted against 50 kinases, carefully selected to represent the human kinome. Illustration reproduced courtesy of Cell Signaling Technology, Inc. (www.cellsignal.com)

Table S1. Crystallography: Data collection and refinement statistics

	hGWL-KinDom
**Data collection**	
Space group	$P2_1\,2_1\,2_1$
Cell dimensions	
a, *b*, *c* (Å)	42.90, 80.28, 179.9
α, β, γ (°)	90, 90, 90
Resolution (Å)	48.05 – 3.10 (3.18-3.10) *
R_{pim}	0.122 (0.673)
Mn I / σI	7.3 (1.5)
$CC^1/_2$	0.985 (0.497)
Completeness (%)	99.5 (99.8)
Multiplicity	4.4 (4.5)
**Refinement**	
Resolution (Å)	48.05 – 3.1
No. reflections	21632
R_{work} / R_{free}	0.18, 0.26
No. atoms	
Protein	3834
Ligand/ion	87
Water	58
B-factors	
Protein	59.34
Ligand/ion	57.94
Water	45.75
R.m.s. deviations	
Bond lengths (Å)	0.010
Bond angles (°)	1.377
**Molprobity** (version 4.2)	
MolProbity Score	2.58 (94th percentile)
Ramachandran favoured	429, 87.73%
Ramachandran outliers	14, 2.86%

*Values in parentheses are for highest-resolution shell

Table S2. Pairwise distance alignment search of GWL ligand-binding site

Kinase:	Pairwise Distance	Drugs	Bioactivities	Compounds
STK38	0.65	0	17	17
STK38L	0.65	0	97	75
ROCK1	0.85	1	756	579
PKC_z	1.54	0	890	798
PKC_i	1.56	0	653	579

References

1. Kabsch W (2010) Xds. *Acta Crystallogr D Biol Crystallogr* 66(Pt 2):125-132.
2. Evans P (2006) Scaling and assessment of data quality. *Acta crystallographica Section D, Biological crystallography* 62(Pt 1):72-82.
3. Evans PR (2011) An introduction to data reduction: space-group determination, scaling and intensity statistics. *Acta crystallographica Section D, Biological crystallography* 67(Pt 4):282-292.
4. Evans PR & Murshudov GN (2013) How good are my data and what is the resolution? *Acta Crystallogr D Biol Crystallogr* 69(Pt 7):1204-1214.
5. Winn MD*, et al.* (2011) Overview of the CCP4 suite and current developments. *Acta Crystallogr D Biol Crystallogr* 67(Pt 4):235-242.
6. McCoy AJ*, et al.* (2007) Phaser crystallographic software. *J Appl Crystallogr* 40(Pt 4):658-674.
7. Biondi RM*, et al.* (2002) High resolution crystal structure of the human PDK1 catalytic domain defines the regulatory phosphopeptide docking site. *EMBO J* 21(16):4219-4228.
8. Emsley P & Cowtan K (2004) Coot: model-building tools for molecular graphics. *Acta Crystallogr D Biol Crystallogr* 60(Pt 12 Pt 1):2126-2132.
9. Adams PD*, et al.* (2010) PHENIX: a comprehensive Python-based system for macromolecular structure solution. *Acta Crystallogr D Biol Crystallogr* 66(Pt 2):213-221.
10. Brown NR*, et al.* (1999) Effects of Phosphorylation of Threonine 160 on Cyclin-dependent Kinase 2 Structure and Activity. *The Journal of Biological Chemistry* 274(13):8746 - 8756.
11. Mezna M*, et al.* (2012) Development of a high-throughput screening method for LIM kinase 1 using a luciferase-based assay of ATP consumption. *J Biomol Screen* 17(4):460-468.
12. Worzella T & Gallagher A (2007) Optimizing Kinase Assays for Ultrahigh-Throughput Profiling using the Kinase-Glo plus Assay. *Journal of Laboratory Automation* 12(2):99-103.
13. Mohamed S, Rizvi D, Shakil S, & Haneef M (2013) A SIMPLE CLICK BY CLICK PROTOCOL TO PERFORM DOCKING: AUTODOCK 4.2 MADE EASY FOR NON-BIOINFORMATICIANS. *EXCLI Journal* 12:831 - 857.
14. Hegarat N*, et al.* (2014) PP2A/B55 and Fcp1 regulate Greatwall and Ensa dephosphorylation during mitotic exit. *PLoS Genet* 10(1):e1004004.
